# Targeting the epigenetic readers in Ewing Sarcoma inhibits the oncogenic transcription factor EWS/Fli1

**DOI:** 10.18632/oncotarget.8214

**Published:** 2016-03-19

**Authors:** Camille Jacques, François Lamoureux, Marc Baud’huin, Lidia Rodriguez Calleja, Thibaut Quillard, Jérôme Amiaud, Franck Tirode, Françoise Rédini, James E. Bradner, Dominique Heymann, Benjamin Ory

**Affiliations:** ^1^ INSERM, UMR 957, Équipe Labellisée Ligue 2012, Nantes, France; ^2^ Physiopathologie de la Résorption Osseuse et Thérapie des Tumeurs Osseuses Primitives, Université de Nantes, Nantes Atlantique Universités, EA3822, Nantes, France; ^3^ Nantes University Hospital, Nantes, France; ^4^ Institut Curie, INSERM U830, Paris, France; ^5^ Department of Medical Oncology, Dana-Farber Cancer Institute, Harvard Medical School, Boston, Massachusetts, USA; ^6^ Department of Medicine, Harvard Medical School, Boston, Massachusetts, USA

**Keywords:** Ewing Sarcoma, JQ1, bromodomain, epigenetic, EWS/Fli1

## Abstract

Ewing Sarcoma is a rare bone and soft tissue malignancy affecting children and young adults. Chromosomal translocations in this cancer produce fusion oncogenes as characteristic molecular signatures of the disease. The most common case is the translocation t (11; 22) (q24;q12) which yields the EWS-Fli1 chimeric transcription factor. Finding a way to directly target EWS-Fli1 remains a central therapeutic approach to eradicate this aggressive cancer. Here we demonstrate that treating Ewing Sarcoma cells with JQ1(+), a BET bromodomain inhibitor, represses directly EWS-Fli1 transcription as well as its transcriptional program. Moreover, the Chromatin Immuno Precipitation experiments demonstrate for the first time that these results are a consequence of the depletion of BRD4, one of the BET bromodomains protein from the EWS-Fli1 promoter. *In vitro*, JQ1(+) treatment reduces the cell viability, impairs the cell clonogenic and the migratory abilities, and induces a G1-phase blockage as well as a time- and a dose-dependent apoptosis. Furthermore, in our *in vivo* model, we observed a tumor burden delay, an inhibition of the global vascularization and an increase of the mice overall survival. Taken together, our data indicate that inhibiting the BET bromodomains interferes with EWS-FLi1 transcription and could be a promising strategy in the Ewing tumors context.

## INTRODUCTION

Ewing Sarcoma is the second most common bone cancer after Osteosarcoma. It is nonetheless an infrequent tumor, mostly affecting children and young adults, with a peak of incidence around 15 years [1]. Described for the first time in 1921 by James Ewing [2], it mainly arises at the pelvic bones, the diaphysis of the lower extremities’ long bones and the chest wall's bones. The standard of care for young patients suffering from Ewing Sarcoma is based on a multimodal therapy including surgical resection associated with local radiotherapy and chemotherapy combination [1, 3–5]. This strategy has markedly improved the patient outcome worldwide, since the 5-year survival rates after treatment are currently around 60–70% for the localized forms. Nonetheless, these rates dramatically fall around 20–40% in metastatic diseases, depending on the metastatic sites and burden [6]. The unsatisfactory prognosis of such patients and the fact that the toxicity thresholds have already been achieved underscore the necessity to find novel therapeutic strategy.

Since 1992, this cancer is well characterized by the reciprocal translocation t (11;22) (q24;q12), found in 85% of these tumors. This translocation leads to the gene fusion between the 5′ segment of the *EWSR1* gene (Ewing sarcoma breakpoint region 1) on the chromosome 22 and the 3′ portion of *Fli1* (Friend leukemia virus integration site 1), located on the chromosome 11 [7]. It gives birth to the chimeric protein EWS-Fli1, which behaves as an aberrant transcription factor at the origin of the tumorigenic potential of Ewing Sarcoma cells [8]. EWS-Fli1 indeed acts as a pivotal transcriptional modulator, up-regulating target genes such as c-myc [9–11], ID2 [11], Cyclin D1 (CCND1) [11, 12], Gli1 [13], MMP-3 [14], VEGFA [15, 16], NR0B1 [17, 18], FOXM1 [19] or EZH2 [20], implicated in cell cycle, invasion and proliferation pathways. EWS-Fli1 has also a transcriptional repressor role [21], as it is able to directly inhibit the expression of some tumor suppressor genes such as p21 [22], p57^kip [10]^, TGF-βRII [11, 21, 23] and IGFBP3 [24], often associated with development, differentiation and cell communication. As Ewing Sarcoma development seems to depend on one specific oncogene, targeting its transcription appears as an attractive strategy. Moreover, RNA interference studies already demonstrated the relevance of this therapeutic strategy [25, 26]. Very little is known about the upstream epigenetic regulation of EWS-Fli1. A better understanding of this fusion-oncogene transcriptional regulation could represent an innovative road to improve patient's clinical outcome.

Over the past years, the bromodomain and extra-terminal domain (BET) proteins have emerged as an important class of epigenetic readers. This protein subfamily encompasses BRD2, BRD3, BRD4 and BRDT and displays gene transcription modulation features by its ability to recognize and bind to the N-acetylated-lysine residues on histone tails [27]. BET bromodomains consequently induce an opened-chromatin structure and act as scaffold proteins to recruit transcriptional complexes and RNA polymerases [28]. The BET proteins are widely associated with cancer progression, as it was demonstrated that BRD4 has the ability to associate with *positive transcription elongation factor b* (P-TEF-b) to promote the G1-S transition of the cell cycle [29]. In addition, the well-known *c-myc* oncogene, which is overexpressed in various cancers, is directly activated by BRD4 [30, 31]. Therefore, several studies report the benefits of targeting the BET bromodomains in cancer [32–34]. In Osteosarcoma, BET bromodomain inhibition impairs *RUNX2* expression [31], a pivotal osteoblastic- and tumorigenic-related gene [35, 36]. From this perspective, the small cell-permeable thieno-triazolo-1, 4-diazepine, JQ1, which is a potent BET inhibitor, has recently been shown to exhibit anti-cancer effects in various cancer models such as NMC (NUT midline carcinoma) [37], hematopoietic malignancies [38] lung cancer [39], prostate cancer [40] and Osteosarcoma [31]. Moreover, strong promoters and super-enhancer regulatory regions are frequently found to control oncogenes expression [41, 42]. Thus, JQ1 was shown to reduce the transcription of such genes, whose expression is more sensitive to the BET bromodomains presence [41]. Regarding the crucial role of EWS-Fli1 in Ewing Sarcoma, we hypothesize that its expression may be controlled by such super-enhancers and consequently activated by the BET bromodomain activity.

In this study, we explore the role of BET bromodomains in the carcinogenesis of Ewing Sarcoma in order to evaluate the therapeutic potential of inhibiting its epigenetic reading activity both *in vitro* and *in vivo*. We further uncover that through targeting EWS-Fli1, the BET bromodomain inhibitor JQ1 consequently modulates the expression level of the downstream EWS-Fli1 network genes in Ewing sarcoma cell lines and in *in vivo* tumor models. The JQ1-mediated transcriptional silencing of EWS-Fli1 corresponds with the release of BRD4 from its loci, which further supports the direct transcriptional activation of this gene by BRD4. Taken together and considering the EWS-Fli1 oncogene addiction of the Ewing Sarcoma cells, our findings indicate that targeting the BET bromodomain signaling pathway in Ewing tumor can effectively disrupt the progression of this cancer through transcriptional repression of its main oncogenic driver [43]. In particular, these results provide a novel glimpse at the importance of epigenetic mechanisms in cancer biology as well as a strong clinical rationale for the use of BET bromodomain inhibitors such as JQ1, as a potent therapeutic approach for Ewing Sarcoma.

## RESULTS

### Ewing Sarcoma cell lines are sensitive to BET proteins inhibition therapy

**Figure 1:**
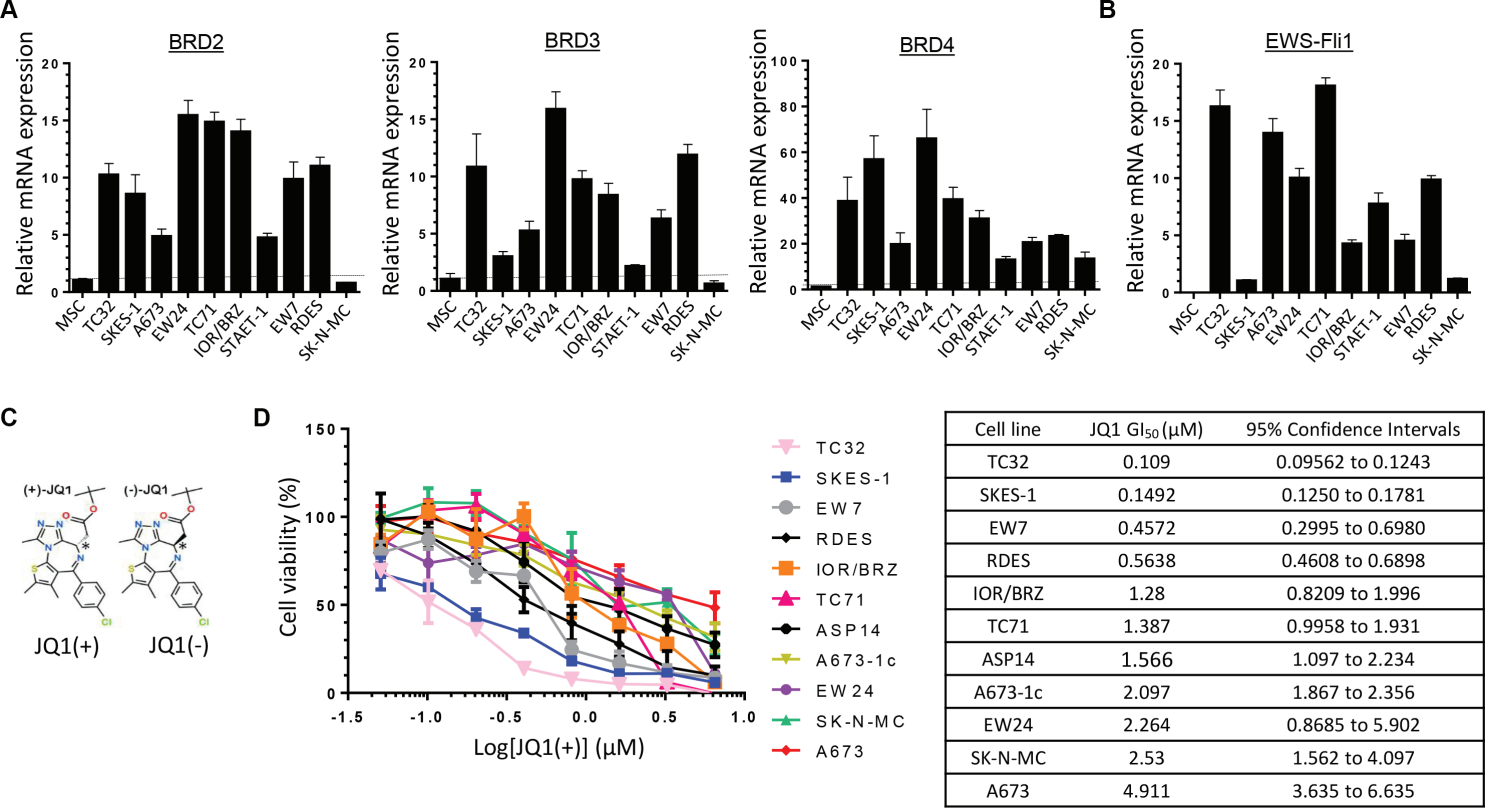
Ewing Sarcoma cell lines overexpress BRD2, BRD3, BRD4 and EWS-Fli1 at mRNAs level and are sensitive to BET proteins inhibition (**A**) Expression of BRD2, BRD3, BRD4 and (**B**) EWS-Fli1 at mRNA levels was evaluated by qRT–PCR in human Ewing Sarcoma cell lines compared with human mesenchymal stem cells (MSCs). GAPDH and B2M are used as housekeeping genes. Error bars show standard deviation for *n* = 3 measurements from representative experiments. (**C**) Chemical structure of the two JQ1 enantiomers. (**D**) Human Ewing Sarcoma cell lines (A673, A673-1c, ASP14, EW7, EW24, IOR/BRZ, RDES, SKES-1, SK-N-MC, TC32 and TC71) were cultured for 48 hours in the presence of JQ1(+) at the indicated concentrations and cell growth was determined by WST-1 assay and compared with control (left panel). GI_50_ for JQ1(+) in tumor cell lines (right panel). These experiments were repeated at least twice. Error bars show standard deviation for *n* = 3 measurements from representative experiments.

To directly assess the relevance of targeting BET bromodomain proteins signaling in Ewing Sarcoma disease, we first evaluated the messenger RNA expression level of BRD2, BRD3 and BRD4 in ten human Ewing Sarcoma cell lines. All the cell lines tested expressed BRD2, BRD3 and BRD4 at least two times more than normal mesenchymal stem cells (MSCs), except the SK-N-MC cells, potentially because of their neuroblastoma origin (Figure 1A). These first data suggest that the majority of the Ewing Sarcoma cells express the required molecular targets for an efficient BET proteins’ inhibition therapeutic strategy. Since the aim of this work is to elucidate the potential implication of the BET bromodomains proteins on the Ewing Sarcoma's carcinogenesis through their possible regulatory role in EWS-Fli1 expression, the EWS-Fli1 mRNA expression level was also assessed (Figure 1B). This screening validated the presence of the fusion oncogene in all the cell lines tested.

To evaluate the therapeutic potential of BET bromodomain inhibition in Ewing Sarcoma, we used the JQ1(+) active form of the soluble pharmacological inhibitor JQ1, a thienotriazolo-1, 4-diazepine that binds selectively to the acetyl-lysine-binding pocket of the BET bromodomain protein (Figure 1C) [44]. The so-called JQ1(−), the JQ1 inactive enantiomer does not harbor such acetyl-lysine-binding pocket selectivity, and will be further used as a negative control. A panel of eleven genetically defined human Ewing Sarcoma cell lines was treated with the BET inhibitor JQ1(+) to assess its effect on cell viability (Figure 1D). A dose-dependent inhibition of cell viability was observed in all the cell lines studied although with significant variability. The TC32, SKES-1, EW7 and RDES cells are the more sensitive ones as they exhibited a GI_50_ between 0.109 and 0.564 μM. With GI_50_ between 1.28 and 2.264 μM, the IOR/BRZ, TC71, ASP14, A673-1c, EW24 and SK-N-MC cells display an intermediate sensitivity. In contrast, the A673 cell line is about 5 times less sensitive compared to the first ones, with a GI_50_ of 4.911 μM (Figure 1D). The JQ1(−) inactive enantiomer does not show any effect on the cell viability (Supplementary Figure S1), confirming the specificity of JQ1(+) towards the BET bromodomains. Following investigations were performed with the A673 and TC71 cell lines because of their differences in terms of BET bromodomains levels and JQ1(+) sensitivities reflecting the heterogeneity of our Ewing Sarcoma cell lines. Moreover, those two cell lines can be used for mouse *in vivo* model and represent two different classical anatomical Ewing sarcoma locations. The A673 cells come from a female donor of 15 years old presenting a tumor localized on muscle whereas the TC71 cells come from a 22 years old male patient, displaying a recurrent humerus sarcoma.

### JQ1 inhibits the clonogenicity, the migratory potential and induces both a G1-phase cell cycle arrest and the apoptosis of human Ewing Sarcoma cell lines

**Figure 2:**
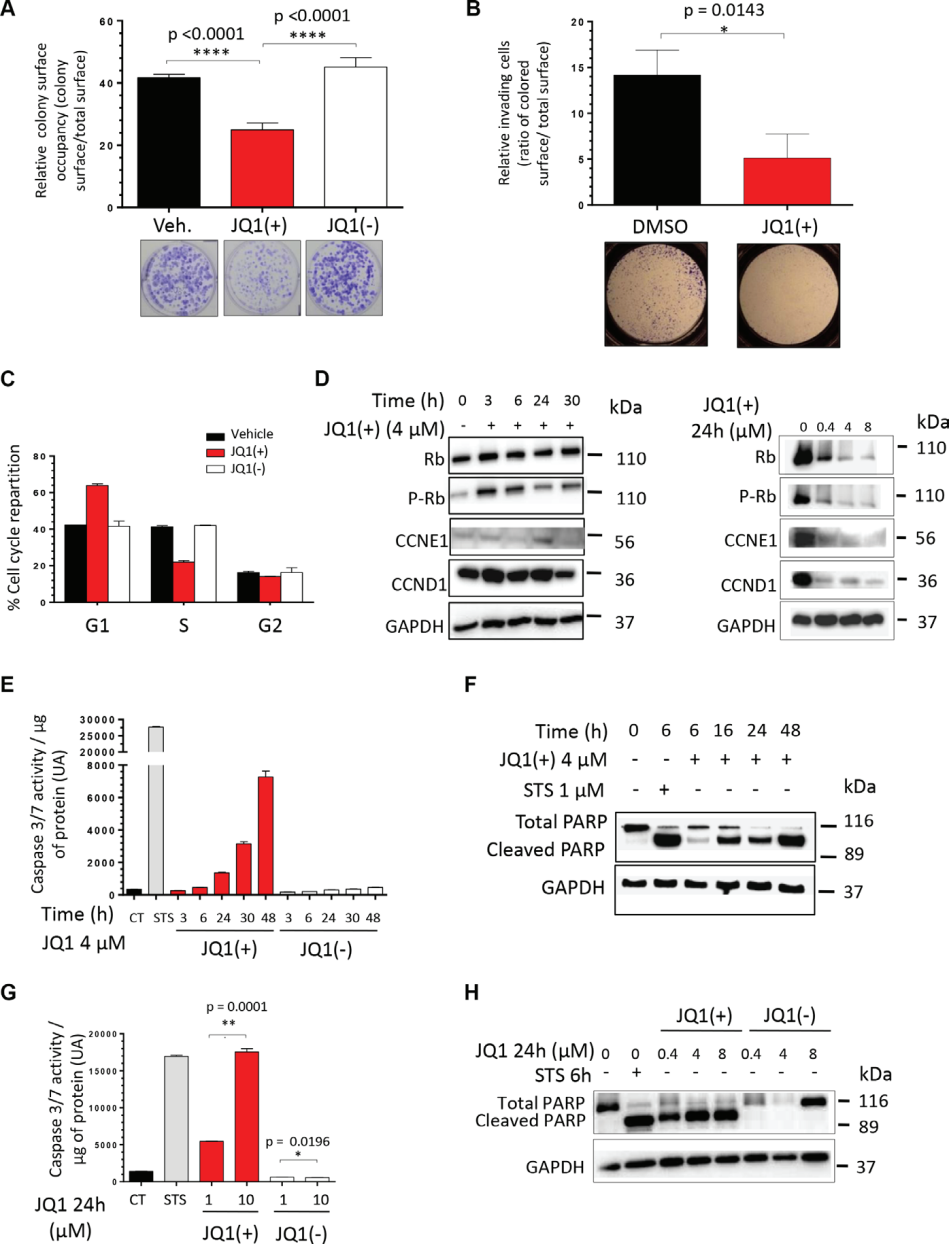
JQ1 inhibits the clonogenicity, the migratory potential and induces both a G1-phase cell cycle arrest and the apoptosis of human Ewing Sarcoma cell lines (**A**) The TC71 Ewing Sarcoma cell line was plated at clonal density for colony numeration and treated with 4 μM JQ1(+) for 48 hours. Colony number was counted thanks to crystal violet staining performed after a 6-days incubation time and pictures of representative wells were taken. These experiments were repeated at least twice. Error bars shows standard deviation for *n* = 3 measurements from representative experiments. A two-tailed paired Student's *t*-test was used to compare the different conditions in the clonogenic assays. (**B**) The TC71 cells were cultured or not in presence of 4 μM JQ1(+) during 24 hours and were then plated in Boyden Chambers, always in presence of JQ1(+) for additional 48 hours. Error bars show the standard deviation for *n* = 8 measurements from representative experiments and a two-tailed paired Student's *t*-test was used to compare the different conditions. (**C**) The TC71 Ewing Sarcoma cell line was treated with 1 μM JQ1(+) for 48 hours and the proportion of cells in G1, S, and G2 phase was determined by propidium iodide staining. (**D**) The TC71 Ewing Sarcoma cell line was treated with 4 μM JQ1(+) for 3, 6, 24 or 30 hours and the cell cycle-related proteins Rb, phosphorylated-Rb, CCNE1and CCND1 expression levels were evaluated by Western blotting (left panel). The expression of the same proteins was assessed after treating or not the cells with JQ1(+) at 0.4, 4 or 8 μM during twenty-four hours (right panel). (**E**) TC71 cells were treated with 4 μM of JQ1(+) or JQ1(−) for 3, 6, 24, 30 or 48 hours, and the apoptosis was evaluated by dosage of the caspase 3/7 activities. (**F**) The TC71 cell line was treated with 4 μM JQ1(+) for 6, 16, 24 or 48 hours and apoptosis was evaluated by cleaved poly (ADP-ribose) polymerase (PARP) level by Western blotting. (**G**) The apoptosis was evaluated by dosage of the caspase 3/7 activity in the TC71 Ewing Sarcoma cell line after JQ1(+) or JQ1(−) treatment at 1 or 10 μM during 24 hours. (**H**) The same cell line was treated with 0.4, 4 or 8 μM JQ1(+) or JQ1(−) for 24 hours and apoptosis was evaluated by cleaved poly (ADP-ribose) polymerase (PARP) level by Western blotting. For all the Western blotting experiments, the Glyceraldehyde-3-phosphate dehydrogenase was used as a loading control. These experiments were repeated at least twice. For the caspase 3/7 activity assays, error bars shows standard deviation for *n* = 3 measurements from representative experiments. A two-tailed paired Student's *t*-test was used to compare the different conditions in these assays. For all the apoptosis assessment assays, a 6 hours Staurosporine (STS) treatment at 1 μM is used as a positive control.

In order to evaluate the functional effects of the BET proteins inhibition *in vitro*, we performed a colony formation assay in presence of DMSO alone, JQ1(+) or JQ1(−). A 2-days treatment with 4 μM of JQ1(+) significantly reduces the clonogenic abilities by 48% in TC71 (Figure 2A) and 45% in A673 cells (Supplementary Figure S2A) compared to the same amount of DMSO alone or the same concentration of JQ1(−). Boyden Chambers assays also reveals that inhibiting the BET proteins significantly reduces the migratory potential of these cell lines (Figure 2B and Supplementary Figure S2B). To complete this functional study, we performed a cell cycle analysis through the propidium iodide staining method after a 2-days JQ1 treatment. A 1 μM JQ1(+) treatment blocks the proliferation of the TC71 cells, through a marked G1 arrest (Figure 2C), whereas this blockage was less important in the A673 ones, a significant G2 phase could be observed (Supplementary Figure S2C). Neither the DMSO alone, nor JQ1(−) have any effect on the cell cycle repartition. The differences obtained between the two cell lines could probably be due to the cell-relative sensitivity to JQ1(+), the A673 displaying a higher GI_50_ than the TC71. In order to better characterize the G1 arrest highlighted, Western blot analysis of the cell-cycle-related proteins was assessed (Figure 2D). The time-course experiment performed during 3, 6, 24 or 30 hours with 4 μM of JQ1(+) shows that the phosphorylated form of the Rb protein (P-Rb), not inhibiting E2F, is shortly activated after 3 and 6 hours of JQ1(+) treatment and then reduced after 24 hours in the TC71, whereas the total Rb protein expression level remains stable for TC71. The same results were obtained in the A673 cells after 30 hours of JQ1(+) treatment (Supplementary Figure S2D). This dephosphorylation of Rb is explained by the proteins levels of both the Cyclin E1 (CCNE1) and the Cyclin D1 (CCND1) that decrease at 30 hours of JQ1(+) treatment, principally in the A673 cells (Figure 2D and Supplementary Figure S2D). A decrease in P-RB and the expression of these cell-cycle-related proteins was also demonstrated in a dose-response experiment in both cell lines with first effects observable with as little as 0.4 μM (Figure 2D and Supplementary Figure S2D).

Reduction of the cell viability and cell cycle arrest are well-known dying-cell features. Thus we finally wondered if the BET bromodomain inhibition mediated by JQ1(+) could result in a cell death induction. To assess the caspases-dependent cell death induced by JQ1(+), both the caspase 3/7 activity and the level of cleaved poly (ADP-ribose) polymerase (PARP) were evaluated after a JQ1 treatment. Consistent with the results of the cell death manual counting and the study from Hensel et al., the caspase 3/7 activity and the PARP cleavage reveal a time-dependent induction of apoptosis in the cell lines tested (Figure 2E, 2F and Supplementary Figure S2E, S2F) [45]. In addition, the same methods also confirm that JQ1(+) induces a dose-dependent apoptosis both in the TC71and the A673 cell lines (Figure 2G, 2H and Supplementary Figure S2G, S2H). On the contrary, neither time- nor dose-dependent apoptosis is induced by a JQ1(−) treatment (Figure 2E, 2G, 2H and Supplementary Figure S2E, S2G, S2H, S2K). The BET bromodomains inhibition-induced apoptosis was thus confirmed both in A673 and in TC71 to be caspase-3/7-dependent. Finally, confirmation of cell death induction was obtained when TC71 and A673 cells were treated with 4 μM JQ1(+) or JQ1(−) during 3, 6, 24, 30 or 48 hours and a time-dependent induction of cell death could be observed when the number of dead cells was evaluated by manual counting after Trypan blue exclusion (Supplementary Figure S2I, S2J).

### Direct transcriptional regulation of EWS-Fli1 by BET proteins

**Figure 3:**
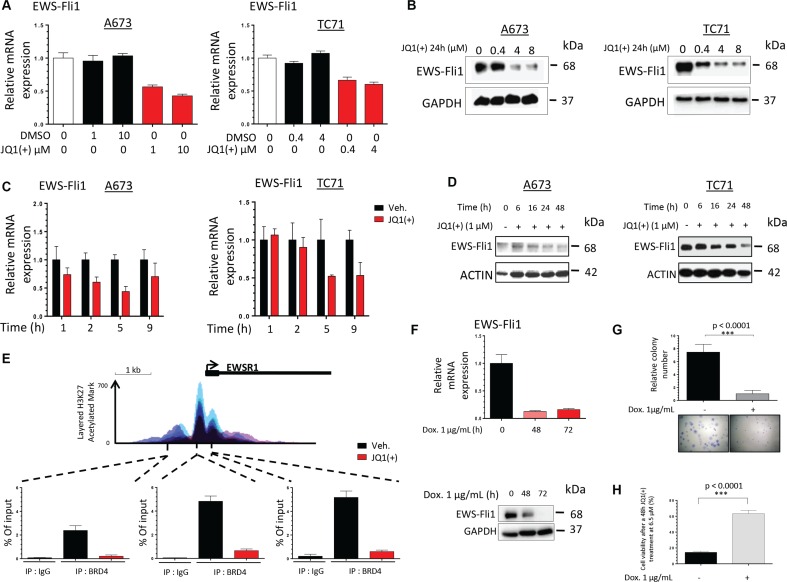
JQ1(+) treatment in Ewing Sarcoma cells induces a dose- and a time-dependent EWS-Fli1 down-regulation (**A**) qRT–PCR for EWS-Fli1 RNA levels in DMSO or JQ1(+)-treated A673 and TC71 Ewing Sarcoma cell lines at different doses for 24 h. GAPDH and B2M expression are used as housekeeping genes. Error bars show standard deviation for *n* = 3 measurements from representative experiments. (**B**) The A673 (left panel) and TC71 (right panel) Ewing Sarcoma cell lines were treated with 0.4, 4 or 8 μM JQ1(+) for 24 hours and the EWS-Fli1 expression level was evaluated by Western blotting. Glyceraldehyde-3-phosphate dehydrogenase was used as a loading control. (**C**) qRT–PCR for EWS-Fli1 RNA levels in DMSO, JQ1(+)-treated A673 (left panel) and TC71 (right panel) Ewing Sarcoma cell lines at different time points, from one to nine hours (4 μM JQ1(+)). GAPDH and B2M are used as housekeeping genes. Error bars show standard deviation for *n* = 3 measurements from representative experiments. (**D**) Immunoblotting of the EWS-Fli1 expression in the same cell lines as represented in (**C**) treated by JQ1(+) for 6, 16, 24 or 48 hours at 1 μM. Actin was used as a loading control. (**E**) ChIP with a BRD4 antibody at three sites within the EWS-Fli1promoter region in A673 cells treated with 4 μM JQ1(+) for 5 h 30. Enrichment is shown as the percentage of total input DNA. Error bars show standard deviation for *n* = 3 measurements from representative experiments. The top track show the levels of enrichment of the H3K27Ac histone mark across the genome as determined by a ChIP-seq assay on seven cell lines from ENCODE. (**F**) The EWS-Fli1 expression was assessed at mRNA level in the ASP14 cell line treated or not with 1 μg/mL Doxycycline for 48 or 72 hours, to induce the shEWS-Fli1 transcription. The EWSFli1expression was assessed at mRNA level after qRT-PCR (upper panel) and at protein level after Immunoblotting (lower panel). GAPDH and B2M are used as housekeeping genes in the qRT-PCR assays. Error bars show standard deviation for *n* = 3 measurements from representative experiments. Glyceraldehyde-3-phosphate dehydrogenase was used as a loading control for the Western blotting. (**G**) ASP14 Ewing Sarcoma cells were treated or not with 1 μg/mL Doxycycline during 72 hours to induce the shEWS-Fli1 transcription and plated at clonal density for colony counts thanks to crystal violet staining performed after a 6-days incubation time. For all the clonogenic assays, error bars show the standard deviation for *n* = 6 measurements from representative experiments and a two-tailed paired Student's *t*-test was used to compare the different conditions. (**H**) ASP14 Ewing Sarcoma cells were treated or not with 1 μg/mL Doxycycline during 72 hours to induce the shEWS-Fli1 transcription and 2000 cells per well were seeded in 96-wells plates. The cells were then treated with 6.5 μM JQ1(+) or the same amount of DMSO. The cell viability was assessed after 48 hours by WST-1 assay. Error bars show the standard deviation for *n* = 3 measurements from representative experiments and a two-tailed paired Student's *t*-test was used to compare the different conditions.

EWS-Fli1 expression is considered as the molecular signature of the Ewing Sarcoma cells and it is now well established that inhibiting this chimeric factor source of oncogene addiction impacts the aggressiveness and the cancerous features of these cells [46]. In order to determine if EWS-Fli1 could be the mediator at the origin of the functional effect of JQ1, we accordingly examined its expression after treatment. In all the Ewing Sarcoma cell lines tested, JQ1(+) treatment decreased the EWS-Fli1 mRNA expression in a dose-dependent manner (Figure 3A). Indeed, after twenty-four hours of 10 μM JQ1(+), the mRNA expression was decreased by 57.1% in the A673 cell line and by 39.9% with 4 μM JQ1(+) in the TC71 cell line. The different JQ1(+) treatment concentrations were picked according to the respective cell lines GI_50_ (Figure 1D). The EWS-Fli1 protein expression level was also assessed by Western blotting in these cell lines (Figure 3B) and confirms the RT-qPCR results. Moreover, as illustrated by the relative quantification of the blots, a 0.4 μM JQ1(+) treatment during twenty-four hours reduces by 56.4% the TC71-EWS-Fli1 protein expression whereas a 4 μM JQ1(+) concentration is necessary to obtain the same effect in the A673 cells (Supplementary Figure S3). These results finally support the fact that the TC71 cell line is more sensitive than the A673 to JQ1(+) treatment and corroborate the recently published ones from Hensel et al., performed in the same cell lines [45]. In addition, we have also shown that the BET bromodomains inhibition not only induces a dose-dependent inhibition, but also a time-dependent inhibition of EWS-Fli1 expression. Interestingly, a quick transcriptional inhibition of EWS-Fli1 expression after only one hour of 4 μM JQ1(+) treatment in the A673 and 5 hours in TC71 cells compared to the DMSO treated-cells is observed (Figure 3C). The time-dependent EWS-Fli1 expression impairment is also confirmed at a protein level, for both cell lines (Figure 3D). The rapid transcriptional effect of the BET inhibitor on EWS-Fli1 gene expression suggests the possibility that BET proteins may exert direct activation of the *EWSR1* locus. To determine whether BET proteins bind directly to the EWS-Fli1 locus, we performed chromatin immunoprecipitation (ChIP) studies by using an antibody targeting BRD4 in A673 cells. We detected specific enrichment of BRD4 within the *EWSR1* promoter region at several locations including both upstream and downstream sites relative to the transcriptional start site of the *EWSR1* gene (Figure 3E). Furthermore, treatment of these cells with JQ1(+) induces the release of BRD4 from the promoter-binding sites. The increased enrichment for histone 3 lysine 27 acetylation (H3K27Ac) at the *EWSR1* promoter region indicates active transcription, perhaps associated with super-enhancer region as recently described [41, 47]. Thus, these findings demonstrate that BRD4 is present on the *EWSR1* promoter region and that JQ1(+) is able to deplete BRD4 from the promoter, thus inhibiting EWS-Fli1 transcription in Ewing Sarcoma cell lines.

In order to demonstrate that EWS-Fli1 is the key mediator of the JQ1(+) functional effects observed in Ewing Sarcoma cells, we performed experiments using the doxycycline-inducible shEWS-Fli1 ASP14 cell line. We first confirmed the clonogenic-promoting effect of EWS-Fli1 in our model, highlighting the fact that suppressing EWS-Fli1 expression mimics the BET bromodomains inhibition effects on the Ewing Sarcoma cells clonogenic capabilities (Figure 3F, 3G). In order to check the crucial implication of EWS-Fli1 in the JQ1(+) response, we then assessed the cellular viability of the same cells after a JQ1(+) treatment, expressing or not EWS-Fli1 (Figure 3H). Our results show that under-expressing EWS-Fli1 significantly reduces the sensitivity to a forty-eight hours JQ1(+) treatment at 6.5 μM. Taken together, those data emphasise and support the BET bromodomains implication in the EWS-Fli1 expression activation and supports the fact that JQ1 has an EWS-Fli1-dependent activity in our model.

### Inhibition of the BET proteins modulates the expression of EWS-Fli1 transcriptional-targets in a dose- and time-dependent manner

**Figure 4:**
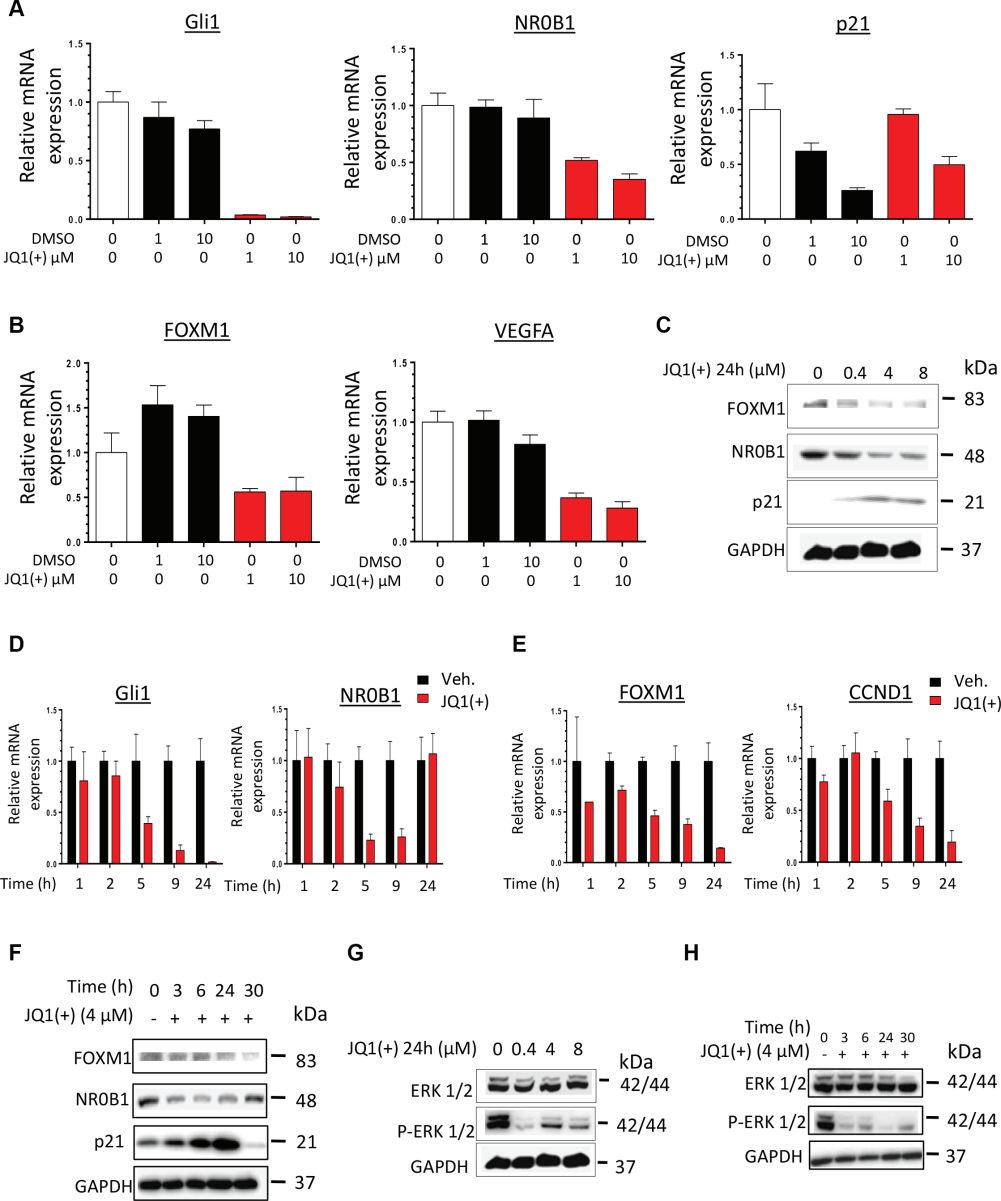
JQ1(+) treatment in Ewing Sarcoma cells induces a dose- and a time-dependent modulation of pathways under the control of EWS-Fli1 (**A**) qRT–PCR for EWS-Fli1 direct-target genes (Gli1, NR0B1 and p21) and (**B**) EWS-Fli1indirect-target genes (FOXM1 and VEGFA) in DMSO or JQ1(+)-treated Ewing Sarcoma A673 cell line during 24 h, at 1 and 10μM JQ1(+). (**C**) FOXM1-, NR0B1- and p21-EWS-Fli1-target gene expression was evaluated at protein level by Immunoblotting in the A673 Ewing Sarcoma cell line after JQ1(+) treatment at 0.4, 4 or 8 μM during 24 h. Glyceraldehyde-3-phosphate dehydrogenase was used as a loading control. (**D**) qRT–PCR for EWS-Fli1 direct target genes (Gli1and NR0B1) and (**E**) EWS-Fli1 indirect-target genes (FOXM1 and CCND1) in DMSO (Veh.) or JQ1- (+)-treated Ewing Sarcoma A673 cells at 4 μM during 1, 2, 5, 9 and 24 hours. (**F**) FOXM1-, NR0B1- and p21-EWS-Fli1-target gene expression was evaluated at protein level by Immunoblotting in the A673 Ewing Sarcoma cell line after JQ1(+) treatment or not during 3, 6, 24, or 30 hours. (**G**) ERK ½ phosphorylation level was evaluated by Immunoblotting in the A673 Ewing Sarcoma cell line after a 24 hours JQ1(+) treatment at 0.4, 4 or 8 μM or after a 4 μM JQ1(+) treatment during 3, 6, 24, or 30 hours (**H**). All the Western blots were performed at least twice and representative blots are presented. Glyceraldehyde-3-phosphate dehydrogenase was used as a loading control. For all the qRT-PCR, GAPDH and B2M are used as housekeeping genes and error bars show standard deviation for *n* = 3 measurements from representative experiments.

To confirm the downstream functional impact of the EWS-Fli1 transcriptional inhibition mediated by JQ1(+), the expression of some majors EWS-Fli1 target genes was evaluated. Thus it is now well established that Gli1 [13, 48], NR0B1 [18] and p21 [22] are *bona-fide* direct-EWS-Fli1 transcriptional targets, whereas the expression of both FOXM1 [19] and VEGFA [15] is reported to be indirectly under EWS-Fli1′s control, as no direct EWS-Fli1 binding at their promoters was currently brought to light. In agreement with what we observed for EWS-Fli1 expression (Figure 3A), a twenty-four hours treatment with JQ1(+) inhibits Gli1 and NR0B1 mRNA expression in the two cell lines tested (Figure 4A and Supplementary Figure S4A). Furthermore, p21, whose expression is normally inhibited by EWS-Fli1, is increased by about 2-fold in these conditions (Figure 4A and Supplementary Figure S4A). In addition, the expression of two of the EWS-Fli1′s indirect target-genes FOXM1 and VEGFA was also reduced consequently to a JQ1(+) treatment (Figure 4B and Supplementary Figure S4B). The same dose-dependent modulation of the EWS-Fli1-targets was confirmed at the protein level (Figure 4C and Supplementary Figure S4C). Furthermore, a time-course treatment with 4 μM JQ1(+) during 1, 2, 5, 9 or 24 hours also highlighted a time-dependent modulation of Gli1, NR0B1, FOXM1 and CCND1 (Figure 4D, 4E and Supplementary Figure S4D, S4E), which is however faster and more pronounced for the EWS-Fli1 direct-targets than the indirect-ones. We observed the same tendency in the JQ1(+)-ability to modulate the aforementioned genes in a time-dependent manner at protein level (Figure 4F and Supplementary Figure S4F).

In order to confirm the EWS-Fli1-dependent modulation of the expression of Gli1, p21, NR0B1, FOXM1 and VEGFA, we assessed the expression of all these genes in the ASP14 cell line, which bears a doxycycline-inducible shEWS-Fli1. EWS-Fli1 knock-down in these cells results in the inhibition of Gli1, NR0B1, FOXM1, VEGFA and CCND1 expression as well as the induction of p21 one (Supplementary Figure S4, S4I, S4J, S4K).

The BET proteins inhibition's effects on EWS-Fli1′s regulated pathways were also assessed. The MAPK/ERK signaling has recently been suggested as a pivotal pathway in Ewing tumor proliferation and malignant progression [49]. Particularly, it was previously reported that the ERK1/2 proteins are constitutively activated in NIH3T3 cells transformed by an artificial expression of EWS-Fli1 [50]. We first confirmed this observation in our model. Indeed, an EWS-Fli1 knockdown in the ASP14 cells consequently reduces the ERK1/2 phosphorylation-marks, witnesses of the MAPK/ERK pathway activation (Supplementary Figure S4L). In accordance with these results, a dose- as well as a time-dependent inhibition of the ERK1/2 signaling was observed in both the TC71 and the A673 cell lines consequently to a JQ1(+) treatment (Figure 4G, 4H and Supplementary Figure S4G, S4H).

To support the EWS-Fli1-related cell cycle-regulation of the Ewing Sarcoma cells, the Rb phosphorylation level as well as the CCNE1 and CCND1 expression were evaluated in the shEWS-Fli1 inducible ASP14 model (Supplementary Figure S4M). A reduction in the Rb phosphorylation level and an inhibition of the CCNE1 and the CCND1 expression were observed, mimicking the BET bromodomain inhibition's effects mediated by JQ1(+) on the cell cycle (Figure 2C, 2D and Supplementary Figure S2C, S2D). The gene expression signature and the modulation of the EWS-Fli1′s related-pathways finally observed after EWS-Fli1 knock-down are in full agreement with the ones resulting from the BET bromodomain inhibition, reinforcing the assertion that JQ1(+) directly inhibits the Ewing Sarcoma malignant features through EWS-Fli1 signaling.

### JQ1 delays Ewing Sarcoma growth and prolongs cancer-specific overall survival

**Figure 5:**
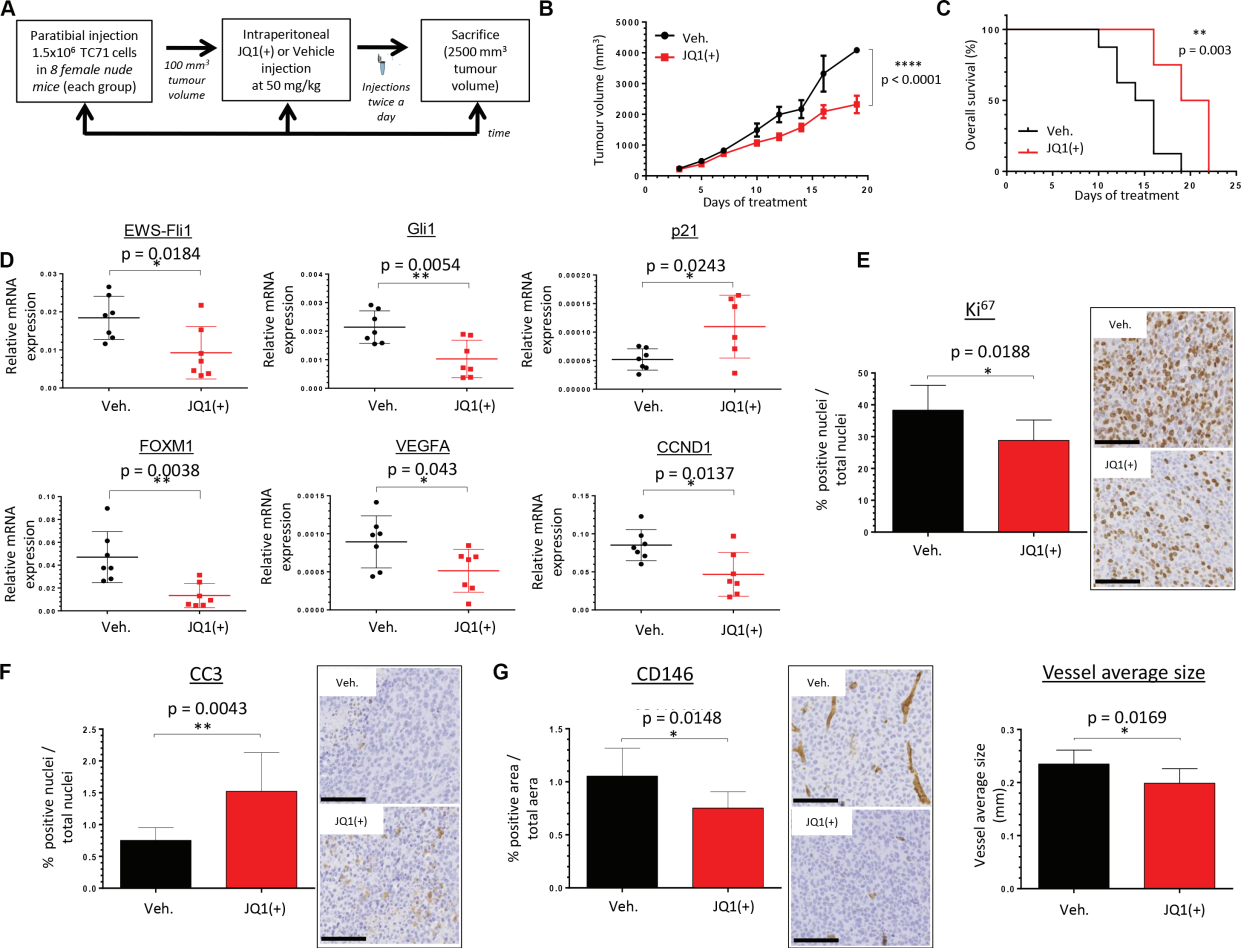
JQ1(+) significantly delays tumor growth in TC71 xenograft model and prolongs cancer-specific survival (**A**) Experimental design: 1.5 million TC71 Ewing Sarcoma cells were injected paratibially in nude mice. Mice were IP injected with 50 mg/kg JQ1(+) or vehicle (10% HP-β-CD) twice a day for the indicated time when tumor volume reached 100 mm^3^. (**B**) The mean tumor volume of mice treated was compared with control group ± s.e.m (*n* = 8 mice in each group).Two-way ANOVA statistical test was used. (**C**) In Kaplan–Meier curves, cancer-specific survival were compared between mice treated with JQ1(+) and control. The log-rank (Mantel-Cox) test was used to compare the overall survival between groups. (**D**) EWS-Fli1, Gli1, p21, FOXM1, VEGFA and CCND1 expressions were evaluated in tumor tissues, after RNA extraction, by qRT–PCR. For all the qRT-PCR, GAPDH and B2M are used as housekeeping genes. Unpaired two-tailed *t*-test was used to compare the gene expression between groups. (**E**) Tumors were collected at the time of euthanasia and Ki67 was evaluated by immunohistochemical analysis (original magnification, 200; scale bar, 100 μm). (**F**) Tumors were collected at the time of euthanasia and caspase 3 activity was evaluated by immunohistochemical analysis (original magnification, 50; scale bar, 250 μm). (**G**) Tumors were collected at the time of euthanasia and CD146 staining was performed by immunohistochemical analysis (left panel). The size of vessels (right panel) was also calculated thanks to the Image J software. For each mouse, ten pictures were taken and were scored and estimated in % of positive cells ± s.d. for DAB activity, in the delimited region of interest (ROI) within the tumor. Error bars show s.d. for *n* = 80 pictures.

To investigate the *in vivo* therapeutic potential of BET inhibition in Ewing Sarcoma, we used a human xenograft model known to recapitulate the features of the human pathology. 1.5 million TC71 tumor cells were implanted in paratibial in athymic mice, and mice were then intraperitoneally (IP) injected with JQ1(+) (50 mg/kg) or vehicle twice a day when the tumor volume reaches 100 mm^3^ (Figure 5A). JQ1(+) treatment significantly reduced the average tumor growth in our model (Figure 5B). Moreover, the Kaplan-Meier analysis revealed that BET proteins inhibition significantly prolonged the overall survival of tumor-bearing mice (Figure 5C). Expression analysis for EWS-Fli1 was performed by qRT-PCR on the mice tumor biopsies. Accordingly to our *in vitro* results, we observed a significant repression of EWS-Fli1 expression in the mice cohort treated with JQ1(+) (Figure 5D). In order to functionally validate the JQ1(+)-mediated transcriptional repression of EWS-Fli1 in our *in vivo* model, we also examined the EWS-Fli1 direct- and indirect-target genes Gli1, p21, FOXM1, VEGFA and CCND1 expression (Figure 5D). As expected, Gli1, FOXM1, VEGFA and CCND1 expression were significantly decreased in the JQ1(+) treated group, whereas the one of p21 was increased. However no statistical change was observed concerning the NR0B1 expression in these conditions (Supplementary Figure S5A).

To better characterize the functional effect of the BET bromodomains inhibition within the tumoral-tissue, immunohistochemical staining for the proliferative marker Ki^67^ was performed. Tumor biopsies from JQ1(+)-treated mice display a statistically significant decreased cell proliferation compared with their untreated counterparts (Figure 5E). In addition, a correlation between the Ki^67^ staining and EWS-Fli1 expression is observed in the tumor samples, (Supplementary Figure S5B). Immunohistochemical staining for the caspase-3 activity was also assessed in these samples and the results show an higher induction of apoptosis in the JQ1(+) cohort (Figure 5F). Because of the *in vitro* decrease of the VEGFA expression observed after inhibition of BET proteins, we determined the JQ1(+) effect on the vascularization within the tumors by performing a CD146 immunohistochemical staining. Different vascularization-parameters were estimated, and JQ1(+) not only inhibits the global vascularization, but also contributes to reduce the average vessels size (Figure 5G). Collectively, these *in vivo* data suggest that the potent anti-tumor activity of JQ1(+) in Ewing Sarcoma is mediated through its inhibitory effects on the EWS-Fli1oncogene expression and its transcriptional targets.

## DISCUSSION

Since EWS-Fli1 was identified as the crucial driver-oncogene in Ewing Sarcomas, it is considered as the best therapeutic target for this pathology. Impairing its activity through a direct inhibition of its transcript is indeed a seducing strategy to reduce the malignant features of the Ewing Sarcoma cells. In this perspective, mithramycin, trabectedin and the so-called YK-4–279, an inhibitor of the EWS-Fli1/RNA helicase A interaction have recently been uncovered as potent EWS-Fli1 transcription factor activity inhibitors [51–53], however without inhibiting the transcription of this oncogene itself. Consequently, novel strategies implicating an early control of its expression, upstream its transcription could be of great interest in this context.

In this study, we highlight the essential implication of BET bromodomain signaling in the control of EWS-Fli1 expression, as well as the potential application of BET bromodomain inhibitors as powerful antitumor agents in Ewing Sarcoma.

The recently identified “super-enhancers” regions are large DNA portions, characterized by both a high density of cell type-specific master transcription factors and a H3K27Ac enrichment [54]. Such enhancers fine-tune the expression of key cell-identity genes and are consequently implicated in the cell fate determination since they allow adaptations of the transcriptional program to the cell environment. Considering both the central role of EWS-Fli1 in Ewing Sarcoma and the cell's addiction to this oncogene, we hypothesized that super enhancers might govern its expression.

We herein demonstrated that treating Ewing Sarcoma cells with JQ1, a BET bromodomain inhibitor reduces their viability, their clonogenic features as well as their migratory capabilities. Moreover, it also leads to a G1-phase arrest, characterized by a P-Rb, a CCND1 and a CCNE1 reduced expression. This cell-cycle effect was reproduced in a shEWS-Fli1 induced model and is in agreement with previously published data reporting the same results down-regulating EWS-Fli1 with antisense oligonucleotides [12]. In addition, we have shown that this disturbance of the cell-cycle ultimately leads to an apoptotic cell-death, mediated by the caspase 3/7 pathway and resulting in the PARP cleavage.

The functional effects of the BET bromodomain inhibition observed in our Ewing Sarcoma models are indeed attributable to the strong reduction in the oncogenic-driver EWS-Fli1 expression, which are both dose- and time-dependent. We indeed demonstrated for the first time through chromatin immunoprecipitation that BRD4 actively binds to the EWS-Fli1 promoter, at H3K27Ac enriched sites. As a consequence, we also showed that a JQ1(+) treatment leads to the release of BRD4 from the EWS-Fli1 promoter. Interestingly, it was recently reported that the H3K27Ac were the histone marks the most strongly inhibited by EWS-Fli1 knockdown [43]. This information sheds light on the possibility that the reduced EWS-Fli1 expression mediated by BET bromodomain inhibitors such as JQ1(+) could also lead to a generalized transcriptional inhibition, as well as having a consequent downstream effect on the EWS-Fli1 controlled-target genes. Our work demonstrated that it reduces in a dose- and in a time-dependent manner the expression of Gli1, NR0B1, FOXM1 and VEGFA, some of the EWS-Fli1 target-genes considered as determining factors in the carcinogenesis of Ewing Sarcoma [19, 55–57]. In addition, we have also demonstrated that the JQ1(+)-mediated BET bromodomain inhibition has a consequent downstream effect on the ERK1/2 signaling, one of the pathways that EWS-Fli1 normally activates and which contributes to the preservation of the Ewing Sarcoma malignant features [49].

The antitumor effects of JQ1(+) on Ewing Sarcoma were also confirmed *in vivo*, as intra-peritoneal administrations of JQ1(+) significantly improve the overall survival of tumor-bearing nude mice through delaying the tumor growth. Our histological analyses point that the cells within the tumors of the treated-animals display a slowed proliferation and are more prone to apoptotic death. The results of our *in vivo* orthotopic model are in full agreement with those of Hensel et al. in their subcutaneous heterotopic Ewing Sarcoma model [45]. In addition, the BET inhibitory treatment leads to a significant reduction of both the global tumor-vascularization as well as the VEGFA expression within the tumor-cells. These results corroborate the decreased expression of this angiogenesis regulator, which is also one of the EWS-Fli1 target genes, observed *in vitro*. Interestingly, VEGF was reported to play an important prognostic role in soft tissue sarcomas and in Ewing tumors, as its expression correlates with a poor patient survival [15, 58].

In conclusion, our results shed light on the BET bromodomains’ role, especially BRD4′s one, as essential regulators of Ewing Sarcoma carcinogenesis. Indeed, we herein bring novel glimpse at their requirement in the control of EWS-Fli1′s expression, the master Ewing Sarcoma-specific oncogene. BET bromodomain protein inhibition consequently impacts EWS-Fli1′s downstream signaling, which is functionally decisive for the interference in the tumorigenic program of these cancer-cells. The work presented in this study provides support for evaluating the BET bromodomain-related protein family as a promising therapeutic target.

## MATERIALS AND METHODS

### Tumor cell lines

Ten human Ewing Sarcoma cell lines were used: the A673 (young 15 years old female Ewing Sarcoma from muscle origin), TC32 (young 31 month female from solid primitive neuroectodermal origin), SKES-1 (young 18 years old Caucasian male Ewing Sarcoma from bone origin), SK-N-MC (young 14 years old Caucasian female from neurogenic origin) and RDES (young 19 years old Caucasian male Ewing Sarcoma from bone origin) cell lines were kindly provided by Dr. S. Burchill (Children's Hospital, Leeds, United Kingdom) and the EW24 and TC71 cell lines by Dr. O. Delattre (Institut National de la Santé et de la Recherche Médicale U830, Paris, France). The A673-1c and the ASP14 (A673/TR/shEF) cells were kind gifts from Dr. F. Tirode (Institut Curie, Paris, France) and F. R. Alonso Garcia de la Rosa (Instituto de Investigacion en Enfermedades Raras, Instituto de Salud Carlos III, Majadahonda, Spain) respectively. The ASP14 cells were cultured in presence of zeocin 50 μg/mL and blasticidin 2 μg/mL, and the shEWS-Fli1 system was induced by adding 1 μg/mL doxycycline. The cell lines A673, A673-1c, ASP14, TC32, SKES-1 and RDES were cultured in Dulbecco's modified Eagle's medium (DMEM; Invitrogen-Life Technologies Inc.) supplemented with 10% fetal bovine serum (FBS; Hyclone) and 2 mmol/L L-glutamine. The SK-N-MC, STAET-1, EW7, EW24, and TC71 cells were cultured in RPMI (Invitrogen-Life Technologies Inc.) with 10% FBS. The IOR/BRZ cells were cultured in Iscove's modified Dulbecco's medium (IMDM Invitrogen-Life Technologies Inc.) with 10% FBS and 2 mmol/L L-glutamine. All cell lines were cultured in the presence of 1% penicillin/streptomycin and type I collagen (Sigma-Aldrich) was required to allow the STAET-1, TC71and EW7 cells to grow. All cell lines were cultured in a humidified 5% CO_2_/air atmosphere at 37°C and were passaged for less than 3 months.

### Therapeutic agents

BRD4 inhibitor, JQ1, was kindly provided by James Bradner (Dana-Farber Cancer Institute). This synthetic compound targets selectively the acetyl-lysine-binding pocket of the BET bromodomain proteins. For *in vitro* studies, JQ1 was dissolved in dimethyl sulphoxide (DMSO) at 10 mM stock solutions and stored at −20°C. For the *in vivo* studies, JQ1 was dissolved in DMSO at 50 mg/ml and then diluted in 10% hydroxypropyl beta cyclodextrin (HP-β-CD, Sigma-Aldrich) to get the final dose, 50 mg/kg and stored at 4°C. The 10% HP-β-CD is prepared in sterile water, which was filtered with 0.22 μm filter. During this study, the active enantiomer of JQ1 was called JQ1(+), whereas the inactive one was called JQ1(−).

### Cell proliferation assay and GI_50_ calculation

Ewing Sarcoma cell lines were plated in 96-wells plates in the appropriate medium with 10% FBS and treated with JQ1 at indicated concentration and time and cell growth was measured using the WST-1 assay (Roche, Mannheim, Germany). At the end of the incubation time, culture medium is removed and replaced by the WST-1 reagent diluted in fresh medium in a 1:10 proportion. After a 7 hours incubation time, the absorbance at 470 nm of each well was measured on a 96-multiwellmicroplate reader (Victor² 1420; PerkinElmer Inc.) and normalized to the average reading of wells containing medium only. Each assay was performed in triplicate. The GI_50_ were calculated thanks to the GraphPad Prism 6 software.

### Quantitative reverse transcription–PCR

Total RNA was extracted from cultured cells or from crushed-xenograft samples using QIAzol Lysis Reagent (QIAGEN) and the miRNeasy Mini Kit (QIAGEN) according to the manufacturer's instructions. Total RNA was reversed transcribed using the Thermo Script RT-PCR System (Life Technologies). Real-time monitoring of PCR amplification of complementary DNA was performed using DNA primers on CFX96 real-time PCR detector system (Bio-Rad, Marnes la Coquette, France) with SYBR PCR Master Mix buffer (Bio-Rad). Target gene expression was normalized to glyceraldehyde 3-phosphate dehydrogenase and β-2 microglobulin levels in respective samples as an internal standard, and the comparative cycle threshold (Ct) method was used to calculate relative quantification of target messenger RNAs. Each assay was performed in triplicate. The primers sequences are the following: B2M FW: 5′-TTCTGGCCTGGAGGCTATC-3′, RV: 5′-TCAGGAA ATTTGA-3′; BRD2 FW: 5′-GCTTTAGGCCCTTCTG GCTT-3′, RV: 5′-ATCATAACCTGTAGGCAGGGC-3′; BRD3 FW: 5′- CTGAAACCCACCACTTTGCG-3′, RV: 5′-GCTCCTCTTTCGACTTGGCT-3′; BRD4 FW: 5′-G CTCAGGAATGTATCCAGGACT-3′, RV: 5′-CCAGAG CTTCTGCCATTAAGA-3′; BRDT FW: 5′-TGACATCT CTCTCTCGCCCT-3′, RV: 5′-CTCGCCTCTTCACACC CTTT-3′; CCND1 FW: 5′-GCCGAGAAGCTGTGCA TC-3′, RV: 5′-CCACTTGAGCTTGTTCACCA-3′; EWS-Fli1 FW: 5′-GCCAAGCTCCAAGTCAATATAGC-3′, RV: 5′-GGGCCGATTCATGTTATTGC-3′; FOXM1 FW: 5′-GCAGGCTGCACTATCAACAA-3′, RV: 5′-TCGAAG GCTCCTCAACCTTA-3′; GAPDH FW: 5′-TGGGTGTG AACCATGAGAAGTATG-3′, RV: 5′-GGTGCAGGAGG CATTGCT-3′; Gli1 FW: 5′-ACCCGGGGTCTCAAAC TG-3′, RV: 5′-GGCTGACAGTATAGGCAGAGC-3′; NR 0B1 FW: 5′-TGCTCTTTAACCCGGACGTG-3′, RV: 5′-GCGTCATCCTGGTGTGTTCA-3′; p21 FW: 5′-CGA AGTCAGTTCCTTGTGGAG-3′, RV: 5′-CATGGGTTC TGACGGACAT-3′;VEGFA FW: 5′-CCTTGCTGCTCTA CCTCCAC-3′, RV: 5′-CCACTTCGTGATGATTCTGC-3′.

### 2D Clonogenic assay

Ewing Sarcoma cells were seeded at a density of 1000 cells per well in 6-well plate and treated with 4 μM of JQ1(+), JQ1(−) or the corresponding amount of DMSO for 48 hours. Culture medium was then replaced by fresh one and the cells were cultured for additional 6 days. The colonies were then washed in PBS, fixed for 10 min with glutaraldehyde 10% and stained with crystal violet (1% in water) for 10 min at room temperature. Pictures of the stained wells were taken and the stained surfaces/total areas of each well were calculated thanks to the ImageJ software. Each assay was performed in triplicate and two areas per well were randomly chosen to take pictures.

### Boyden chamber assay

Ewing Sarcoma cells were cultured or not in presence of JQ1(+) during 24 hours and 20 000 cells were then plated in 8 μm-pored Boyden Chambers (Falcon), always in presence of JQ1(+), in 1% FBS growth medium for additional 48 hours. A 1%/10% FBS-gradient was generated between the upper and the lower Chamber of the system, to promote the cell migration. At the end of the incubation time, the cells on the upper side of the Chamber were mechanically removed and those on the lower side of the Chamber were fixed with 10% Glutaraldehyde and stained with 0.1% Crystal Violet. Pictures of the Chambers were taken and four different areas were arbitrary chosen to perform quantitative analyses. The ratio « colored surface/total surface » was determined thanks to the ImageJ software. Representative pictures of the Boyden were chosen here. For all the Boyden Chambers experiments, error bars show the standard deviation for *n* = 8 measurements from representative experiments and a two-tailed paired Student's *t*-test was used to compare the different conditions.

### Cell cycle analysis

Ewing Sarcoma cell lines were incubated in the presence of 1 μM JQ1 for 48 h, trypsinized, washed and fixed in 70% ethanol for 30 minutes at 4°C. The cells are then washed, centrifugated and the pellet is resuspended in a phospho-citrate solution (Na_2_HPO_4_ 0.2 M; citric acid 0.1 M, pH 7.5). Cells were centrifugated and resuspended in a PBS solution containing 4 mg/ml Ribonuclease A for 30 min at 37°C. 50 mg/ml propidium iodide was then added to each sample for 20 min at 4°C and the cell cycle distribution was then analysed by flow cytometry (Cytomics FC500; Beckman Coulter, Roissy, France) based on 2 N and 4 N DNA content. Multi Cycle AV DNA Analysis software was used to process the data.

### Western blotting analysis

Samples containing equal amounts of protein (depending on the antibody, 15–75 mg) from lysates of cultured Ewing Sarcoma cell lines underwent electrophoresis on SDS-PAGE and were transferred to polyvinylidenedifluoride (PVDF) membranes. The membranes were blocked in 3% BSA-PBS-0.05% Tween at room temperature for 1 h and blots were probed overnight at 4°C with the following primary antibodies: rabbit anti-Fli1 c-19 sc-356, 1:500; rabbit anti-p21 (c-19) sc-397#DO312, 1:200; rabbit anti-FOXM1 c20 sc-502, 1:100; Santa Cruz Biotechnologies, Santa Cruz, CA; rabbit anti-NR0B1 (DAX1) #13538, 1:1000; rabbit anti-P-44/42 MAPK (T202/Y204) #4370S, 1:2000; rabbit anti-p44/42 #9102S, 1:1000; mouse anti-Rb, 4H1 #9309S, 1:1000; rabbit anti-P-Rb (ser807–811) #9308S, 1:1000; rabbit anti-CCND1#2922S 1:1000; mouse anti-CCNE1 (HE12) #4129S 1:1000; rabbit anti-PARP #9542S, 1:1000; rabbit anti-GAPDH 14c10, 1:2000; Cell Signaling Technologies, Beverly, CA; rabbit anti-actinA5060 (20–33) 1:10 000; Sigma-Aldrich; to detect proteins of interests. After incubation, the membranes were washed three times with washing buffer (PBS containing 0.05% Tween) for 5 min. Membranes were then saturated 1 h with 5% milk-PBS-0.05% Tween (Régilait). Membranes were finally incubated for 1 h with secondary antibodies (goat-anti-rabbit sc-2004 #J1512, 1:10000; donkey-anti-mousesc-2314 #C2012 1:5000; Santa Cruz Biotechnologies, Santa Cruz, CA) at room temperature. Specific proteins were detected using SuperSignal^®^ WestDura Extended Duration Substrate (ThermoScientific, Rockford, USA) and a G-Box (Syngene, Cambridge, UK) after washing. Pictures were analysed thanks to ImageJ software. Actin and Glyceraldehyde-3-phosphate dehydrogenase were used as an internal loading control. The relative quantization of EWS-Fli1 expression (Supplementary Figure S3) was performed thanks to ImageJ software and normalized on the Glyceraldehyde-3-phosphate dehydrogenase expression.

### Cell death assessment

Cellular viability was determined using the Trypan Blue dye exclusion method (Trypan Blue, Eurobio, les Ulis, France; final dye concentration: 0.1% *w*/*v*) and a hemocytometer. The caspase 3/7 activity was assessed thanks to the apo-ONE^®^ Homogeneous caspase-3/7 Assay kit (Promega, Madison, USA) according to the manufacturer's instructions. Each assay was performed in triplicate.

### Quantitative ChIP assay

A ChIP assay was performed by the Magnify ChIP system (Invitrogen-Life Technologies Inc.) using 5 mg of BRD4 antibody (BethylLab) or control rabbit IgG (R & D Systems). Real-time PCR (Bio-Rad) was performed on fragmented DNA using specific primers for the *EWSR1* loci. Primers were designed to amplify sites within each gene locus based on the H3K27 acetylation level. The levels of enrichment of the H3K27Ac histone mark across the genome has been determined by a ChIP-seq assay on seven cell lines from ENCODE web resources. We used the following primers: EWSR1–1 sense 5′-CCGTAAACCTCCTCCTGCAT-3′ and anti-sense 5′-AAGCCCTTCACCCTTGCTAA-3′; EWSR1–2 sense 5′-GCAGTTGTTCTAGTCCGGGT-3′ and anti-sense 5′-CCGCAACTCTTGTCCCAGTC-3′; EWSR1–3 sense 5′-AAGACTGAGTGGAGTTGCCG-3′ and anti-sense 5′-GAAGATTCCAGAACCGGCCC-3′. For the ChIP-qPCR, error bars show standard deviation for *n* = 3 measurements from representative experiments. Each assay was performed in triplicate.

### Animal treatment

All procedures involving mice (their housing in the Experimental Therapeutic Unit at the Faculty of Medicine of Nantes (France) and care, the method by which they were anaesthetized and killed, and all experimental protocols) were conducted in accordance with the institutional guidelines of the French Ethical Committee (CEEA.PdL.06). Mice were anaesthetized by inhalation of a combination of isoflurane/air (1.5%, 1 l/min) and buprenorphine (0.05 mg/kg; Temge'sic, Schering-Plough). Tumor volume was measured three times weekly and tumor volume was calculated by using the formula: length*width*depth*0.5. Data points were expressed as average tumor-volume ± s.e.m. Xenograft models were induced by a paratibial injection of 1.5.10^6^ TC71 cells of 5-week-old female athymic nude mice (Harlan Sprague–Dawley Inc.), leading to a rapidly growing tumor in soft tissue with secondary contiguous bone invasion. Once palpable tumors, mice were randomly assigned to vehicle or JQ1(+). When tumors reached 100 mm^3^, JQ1(+) (50 mg/kg; formulation in 10% HP-β-CD in sterile water) is IP injected twice a day, every day. Each experimental group consisted of eight mice. When tumor volume reached 10% of body weight, mice were killed and tumor biopsy samples were collected for evaluation of mRNA expression by RT-qPCR analyses. Tumors were also harvested for immunohistochemistry analyses.

### Immunohistochemistry

Immunostaining for Ki^67^ was conducted using a primary antibody mouse anti-human Ki^67^ (M7249, 1:100; Dako) on tumor section. Immunostaining for CD146 was conducted using a primary antibody rabbit anti-human CD146 (ab75769, 1:800; Abcam) on tumor section. Immunostaining for Cleaved caspase-3 was conducted using a primary antibody rabbit anti-human Cleaved Caspase-3 (9664, 1:400; Cell Signaling) on tumor section. All analyses were assessed by light microscopy using a DMRXA microscope (Leica). The comparisons of Ki^67^ and CD146 staining intensities were made at ×200 magnifications whereas ones of Cleaved caspase-3 were made at ×50 magnifications. Pictures were taken thanks to the NDP. view 2 Hamamatsu software, and the quantization of the staining intensities was performed with ImageJ. For each histological-slide from the tumor-sample of one mouse, 10 areas were arbitrary chosen to represent the whole tumor section. The ImageJ ImmunoRatio version 1.0c 14.2.2011 plugin was used to carry out the Ki^67^ analysis on these 10 areas. All the histograms presented in the Figures result from the average of the DAB-positive surface counting performed on the 10 areas selected for the eight mice per group. The vessels size assessment is based on a pixel scale and according to the results table of the ImageJ software, it corresponds to the average vessel perimeter.

### Statistical analysis

For each experimental data point, the standard deviation from replicate experiments was calculated as noted in the legends and is shown as error bars. All error bars show standard deviation for at least triplicate measurement from representative experiments. The mean ± s.d was calculated for all groups and compared by two-tailed paired Student's *t*-test or by ANOVA analysis of variance. Error bars from the *in vivo* tumor growth monitoring represent the s.e.m from the mean tumor volume of the mice (*n* = 8 mice in each group). *P* < 0.05 was used as the criteria for statistical significance. For the Supplementary Figure S5B, the Pearson product-moment correlation coefficient (*R*²) was calculated. GraphPad Prism 6 software was used for all statistical analysis.

## SUPPLEMENTARY MATERIALS FIGURES



## References

[1] Bernstein M, Kovar H, Paulussen M, Randall RL, Schuck A, Teot LA, Juergens H (2006). Ewing's sarcoma family of tumors: current management. Oncologist.

[2] Ewing J, James Ewing, Classics in oncology, Diffuse endothelioma of bone (1972). Proceedings of the New York Pathological Society, 1921. CA Cancer J Clin.

[3] Schuck A, Ahrens S, Paulussen M, Kuhlen M, Konemann S, Rube C, Winkelmann W, Kotz R, Dunst J, Willich N, Jurgens H (2003). Local therapy in localized Ewing tumors: results of 1058 patients treated in the CESS 81, CESS 86, and EICESS 92 trials. Int J Radiat Oncol Biol Phys.

[4] Grier HE, Krailo MD, Tarbell NJ, Link MP, Fryer CJ, Pritchard DJ, Gebhardt MC, Dickman PS, Perlman EJ, Meyers PA, Donaldson SS, Moore S, Rausen AR (2003). Addition of ifosfamide and etoposide to standard chemotherapy for Ewing's sarcoma and primitive neuroectodermal tumor of bone. N Engl J Med.

[5] Paulussen M, Craft AW, Lewis I, Hackshaw A, Douglas C, Dunst J, Schuck A, Winkelmann W, Kohler G, Poremba C, Zoubek A, Ladenstein R, van den Berg H (2008). Results of the EICESS-92 Study: two randomized trials of Ewing's sarcoma treatment—cyclophosphamide compared with ifosfamide in standard-risk patients and assessment of benefit of etoposide added to standard treatment in high-risk patients. J Clin Oncol.

[6] (2014). Bone sarcomas: ESMO Clinical Practice Guidelines for diagnosis, treatment and follow-up. Ann Oncol.

[7] Delattre O, Zucman J, Plougastel B, Desmaze C, Melot T, Peter M, Kovar H, Joubert I, de Jong P, Rouleau G (1992). Gene fusion with an ETS DNA-binding domain caused by chromosome translocation in human tumours. Nature.

[8] May WA, Lessnick SL, Braun BS, Klemsz M, Lewis BC, Lunsford LB, Hromas R, Denny CT (1993). The Ewing's sarcoma EWS/FLI-1 fusion gene encodes a more potent transcriptional activator and is a more powerful transforming gene than FLI-1. Mol Cell Biol.

[9] Bailly RA, Bosselut R, Zucman J, Cormier F, Delattre O, Roussel M, Thomas G, Ghysdael J (1994). DNA-binding and transcriptional activation properties of the EWS-FLI-1 fusion protein resulting from the t (11; 22) translocation in Ewing sarcoma. Mol Cell Biol.

[10] Dauphinot L, De Oliveira C, Melot T, Sevenet N, Thomas V, Weissman BE, Delattre O (2001). Analysis of the expression of cell cycle regulators in Ewing cell lines: EWS-FLI-1 modulates p57KIP2and c-Myc expression. Oncogene.

[11] Fukuma M, Okita H, Hata J, Umezawa A (2003). Upregulation of Id2, an oncogenic helix-loop-helix protein, is mediated by the chimeric EWS/ets protein in Ewing sarcoma. Oncogene.

[12] Matsumoto Y, Tanaka K, Nakatani F, Matsunobu T, Matsuda S, Iwamoto Y (2001). Downregulation and forced expression of EWS-Fli1 fusion gene results in changes in the expression of G (1) regulatory genes. Br J Cancer.

[13] Beauchamp E, Bulut G, Abaan O, Chen K, Merchant A, Matsui W, Endo Y, Rubin JS, Toretsky J, Uren A (2009). GLI1 is a direct transcriptional target of EWS-FLI1 oncoprotein. J Biol Chem.

[14] Braun BS, Frieden R, Lessnick SL, May WA, Denny CT (1995). Identification of target genes for the Ewing's sarcoma EWS/FLI fusion protein by representational difference analysis. Mol Cell Biol.

[15] Fuchs B, Inwards CY, Janknecht R (2004). Vascular endothelial growth factor expression is up-regulated by EWS-ETS oncoproteins and Sp1 and may represent an independent predictor of survival in Ewing's sarcoma. Clin Cancer Res.

[16] Nagano A, Ohno T, Shimizu K, Hara A, Yamamoto T, Kawai G, Saitou M, Takigami I, Matsuhashi A, Yamada K, Takei Y (2010). EWS/Fli-1 chimeric fusion gene upregulates vascular endothelial growth factor-A. Int J Cancer.

[17] Mendiola M, Carrillo J, Garcia E, Lalli E, Hernandez T, de Alava E, Tirode F, Delattre O, Garcia-Miguel P, Lopez-Barea F, Pestana A, Alonso J (2006). The orphan nuclear receptor DAX1 is up-regulated by the EWS/FLI1 oncoprotein and is highly expressed in Ewing tumors. Int J Cancer.

[18] Garcia-Aragoncillo E, Carrillo J, Lalli E, Agra N, Gomez-Lopez G, Pestana A, Alonso J (2008). DAX1, a direct target of EWS/FLI1 oncoprotein, is a principal regulator of cell-cycle progression in Ewing's tumor cells. Oncogene.

[19] Christensen L, Joo J, Lee S, Wai D, Triche TJ, May WA (2013). FOXM1 is an oncogenic mediator in Ewing Sarcoma. PLoS ONE.

[20] Richter GH, Plehm S, Fasan A, Rossler S, Unland R, Bennani-Baiti IM, Hotfilder M, Lowel D, von Luettichau I, Mossbrugger I, Quintanilla-Martinez L, Kovar H, Staege MS (2009). EZH2 is a mediator of EWS/FLI1 driven tumor growth and metastasis blocking endothelial and neuro-ectodermal differentiation. Proc Natl Acad Sci U S A.

[21] Hahm KB, Cho K, Lee C, Im YH, Chang J, Choi SG, Sorensen PH, Thiele CJ, Kim SJ (1999). Repression of the gene encoding the TGF-beta type II receptor is a major target of the EWS-FLI1 oncoprotein. Nat Genet.

[22] Nakatani F, Tanaka K, Sakimura R, Matsumoto Y, Matsunobu T, Li X, Hanada M, Okada T, Iwamoto Y (2003). Identification of p21WAF1/CIP1 as a direct target of EWS-Fli1 oncogenic fusion protein. J Biol Chem.

[23] Im YH, Kim HT, Lee C, Poulin D, Welford S, Sorensen PH, Denny CT, Kim SJ (2000). EWS-FLI1, EWS-ERG, and EWS-ETV1 oncoproteins of Ewing tumor family all suppress transcription of transforming growth factor beta type II receptor gene. Cancer Res.

[24] Prieur A, Tirode F, Cohen P, Delattre O (2004). EWS/FLI-1 silencing and gene profiling of Ewing cells reveal downstream oncogenic pathways and a crucial role for repression of insulin-like growth factor binding protein 3. Mol Cell Biol.

[25] Kovar H (2010). Downstream EWS/FLI1 - upstream Ewing's sarcoma. Genome Med.

[26] Tanaka K, Iwakuma T, Harimaya K, Sato H, Iwamoto Y (1997). EWS-Fli1 antisense oligodeoxynucleotide inhibits proliferation of human Ewing's sarcoma and primitive neuroectodermal tumor cells. J Clin Invest.

[27] Hewings DS, Rooney TP, Jennings LE, Hay DA, Schofield CJ, Brennan PE, Knapp S, Conway SJ (2012). Progress in the development and application of small molecule inhibitors of bromodomain-acetyl-lysine interactions. J Med Chem.

[28] Prinjha RK, Witherington J, Lee K (2012). Place your BETs: the therapeutic potential of bromodomains. Trends Pharmacol Sci.

[29] Yang Z, He N, Zhou Q (2008). Brd4 recruits P-TEFb to chromosomes at late mitosis to promote G1 gene expression and cell cycle progression. Mol Cell Biol.

[30] Bandopadhayay P, Bergthold G, Nguyen B, Schubert S, Gholamin S, Tang Y, Bolin S, Schumacher SE, Zeid R, Masoud S, Yu F, Vue N, Gibson WJ (2014). BET bromodomain inhibition of MYC-amplified medulloblastoma. Clin Cancer Res.

[31] Lamoureux F, Baud’huin M, Rodriguez Calleja L, Jacques C, Berreur M, Redini F, Lecanda F, Bradner JE, Heymann D, Ory B (2014). Selective inhibition of BET bromodomain epigenetic signalling interferes with the bone-associated tumour vicious cycle. Nature communications.

[32] Delmore JE, Issa GC, Lemieux ME, Rahl PB, Shi J, Jacobs HM, Kastritis E, Gilpatrick T, Paranal RM, Qi J, Chesi M, Schinzel AC, McKeown MR (2011). BET bromodomain inhibition as a therapeutic strategy to target c-Myc. Cell.

[33] Dawson MA, Prinjha RK, Dittmann A, Giotopoulos G, Bantscheff M, Chan WI, Robson SC, Chung CW, Hopf C, Savitski MM, Huthmacher C, Gudgin E, Lugo D (2011). Inhibition of BET recruitment to chromatin as an effective treatment for MLL-fusion leukaemia. Nature.

[34] Zuber J, Shi J, Wang E, Rappaport AR, Herrmann H, Sison EA, Magoon D, Qi J, Blatt K, Wunderlich M, Taylor MJ, Johns C, Chicas A (2011). RNAi screen identifies Brd4 as a therapeutic target in acute myeloid leukaemia. Nature.

[35] Komori T (2002). Runx2, a multifunctional transcription factor in skeletal development. J Cell Biochem.

[36] Pande S, Browne G, Padmanabhan S, Zaidi SK, Lian JB, van Wijnen AJ, Stein JL, Stein GS (2013). Oncogenic cooperation between PI3K/Akt signaling and transcription factor Runx2 promotes the invasive properties of metastatic breast cancer cells. J Cell Physiol.

[37] Beesley AH, Stirnweiss A, Ferrari E, Endersby R, Howlett M, Failes TW, Arndt GM, Charles AK, Cole CH, Kees UR (2014). Comparative drug screening in NUT midline carcinoma. Br J Cancer.

[38] Fiskus W, Sharma S, Qi J, Shah B, Devaraj SG, Leveque C, Portier BP, Iyer S, Bradner JE, Bhalla KN (2014). BET protein antagonist JQ1 is synergistically lethal with FLT3 tyrosine kinase inhibitor (TKI) and overcomes resistance to FLT3-TKI in AML cells expressing FLT-ITD. Mol Cancer Ther.

[39] Shimamura T, Chen Z, Soucheray M, Carretero J, Kikuchi E, Tchaicha JH, Gao Y, Cheng KA, Cohoon TJ, Qi J, Akbay E, Kimmelman AC, Kung AL (2013). Efficacy of BET bromodomain inhibition in Kras-mutant non-small cell lung cancer. Clin Cancer Res.

[40] Lochrin SE, Price DK, Figg WD (2014). BET bromodomain inhibitors—a novel epigenetic approach in castration-resistant prostate cancer. Cancer Biol Ther.

[41] Loven J, Hoke HA, Lin CY, Lau A, Orlando DA, Vakoc CR, Bradner JE, Lee TI, Young RA (2013). Selective inhibition of tumor oncogenes by disruption of super-enhancers. Cell.

[42] Hnisz D, Schuijers J, Lin CY, Weintraub AS, Abraham BJ, Lee TI, Bradner JE, Young RA (2015). Convergence of developmental and oncogenic signaling pathways at transcriptional super-enhancers. Mol Cell.

[43] Tomazou EM, Sheffield NC, Schmidl C, Schuster M, Schonegger A, Datlinger P, Kubicek S, Bock C, Kovar H (2015). Epigenome Mapping Reveals Distinct Modes of Gene Regulation and Widespread Enhancer Reprogramming by the Oncogenic Fusion Protein EWS-FLI1. Cell Rep.

[44] Filippakopoulos P, Qi J, Picaud S, Shen Y, Smith WB, Fedorov O, Morse EM, Keates T, Hickman TT, Felletar I, Philpott M, Munro S, McKeown MR (2010). Selective inhibition of BET bromodomains. Nature.

[45] Hensel T, Giorgi C, Schmidt O, Calzada-Wack J, Neff F, Buch T, Niggli FK, Schafer BW, Burdach S, Richter GH (2016). Targeting the EWS-ETS transcriptional program by BET bromodomain inhibition in Ewing sarcoma. Oncotarget.

[46] Hu-Lieskovan S, Heidel JD, Bartlett DW, Davis ME, Triche TJ (2005). Sequence-specific knockdown of EWS-FLI1 by targeted, nonviral delivery of small interfering RNA inhibits tumor growth in a murine model of metastatic Ewing's sarcoma. Cancer Res.

[47] Whyte WA, Orlando DA, Hnisz D, Abraham BJ, Lin CY, Kagey MH, Rahl PB, Lee TI, Young RA (2013). Master transcription factors and mediator establish super-enhancers at key cell identity genes. Cell.

[48] Zwerner JP, Joo J, Warner KL, Christensen L, Hu-Lieskovan S, Triche TJ, May WA (2008). The EWS/FLI1 oncogenic transcription factor deregulates GLI1. Oncogene.

[49] Chandhanayingyong C, Kim Y, Staples JR, Hahn C, Lee FY (2012). MAPK/ERK Signaling in Osteosarcomas, Ewing Sarcomas and Chondrosarcomas: Therapeutic Implications and Future Directions. Sarcoma.

[50] Silvany RE, Eliazer S, Wolff NC, Ilaria RL (2000). Interference with the constitutive activation of ERK1 and ERK2 impairs EWS/FLI-1-dependent transformation. Oncogene.

[51] Grohar PJ, Woldemichael GM, Griffin LB, Mendoza A, Chen QR, Yeung C, Currier DG, Davis S, Khanna C, Khan J, McMahon JB, Helman LJ (2011). Identification of an inhibitor of the EWS-FLI1 oncogenic transcription factor by high-throughput screening. J Natl Cancer Inst.

[52] Erkizan HV, Kong Y, Merchant M, Schlottmann S, Barber-Rotenberg JS, Yuan L, Abaan OD, Chou TH, Dakshanamurthy S, Brown ML, Uren A, Toretsky JA (2009). A small molecule blocking oncogenic protein EWS-FLI1 interaction with RNA helicase A inhibits growth of Ewing's sarcoma. Nat Med.

[53] Grohar PJ, Griffin LB, Yeung C, Chen QR, Pommier Y, Khanna C, Khan J, Helman LJ (2011). Ecteinascidin 743 interferes with the activity of EWS-FLI1 in Ewing sarcoma cells. Neoplasia.

[54] Pott S, Lieb JD (2015). What are super-enhancers?. Nat Genet.

[55] Beauchamp EM, Ringer L, Bulut G, Sajwan KP, Hall MD, Lee YC, Peaceman D, Ozdemirli M, Rodriguez O, Macdonald TJ, Albanese C, Toretsky JA, Uren A (2011). Arsenic trioxide inhibits human cancer cell growth and tumor development in mice by blocking Hedgehog/GLI pathway. J Clin Invest.

[56] Sengupta A, Rahman M, Mateo-Lozano S, Tirado OM, Notario V (2013). The dual inhibitory effect of thiostrepton on FoxM1 and EWS/FLI1 provides a novel therapeutic option for Ewing's sarcoma. Int J Oncol.

[57] Kinsey M, Smith R, Lessnick SL (2006). NR0B1 is required for the oncogenic phenotype mediated by EWS/FLI in Ewing's sarcoma. Mol Cancer Res.

[58] Yudoh K, Kanamori M, Ohmori K, Yasuda T, Aoki M, Kimura T (2001). Concentration of vascular endothelial growth factor in the tumour tissue as a prognostic factor of soft tissue sarcomas. Br J Cancer.

