# Supplementary material for: Targeting the epigenetic readers in Ewing Sarcoma inhibits the oncogenic transcription factor EWS/Fli1
Source: Oncotarget. 2016 Mar 19;7(17):24125–40. doi: 10.18632/oncotarget.8214 (PMC5029689; doi:10.18632/oncotarget.8214)
Supplement: Supplementary file 1 [file oncotarget-07-24125-s001.pdf]

Targeting the epigenetic readers in Ewing Sarcoma inhibits the oncogenic transcription factor EWS/Fli1

Supplementary Materials

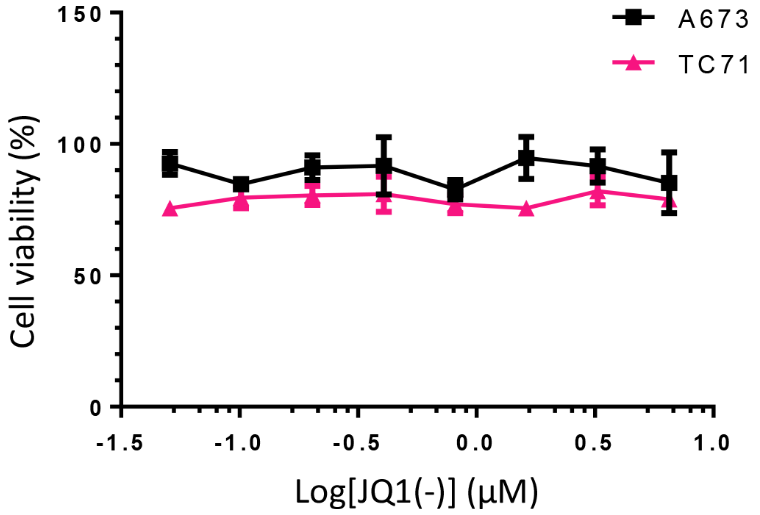

Supplementary Figure S1: The inactive enantiomer JQ1(–) has no effect on the cell viability in Ewing Sarcoma cell lines. TC71 and A673 human Ewing Sarcoma cell lines were cultured for 48 h in the presence of JQ1(–) at the indicated concentrations and cell growth was determined by WST-1 assay and compared with control. These experiments were repeated at least twice. Error bars show standard deviation for n = 3 measurements from representative experiments.

Sup Figure 2

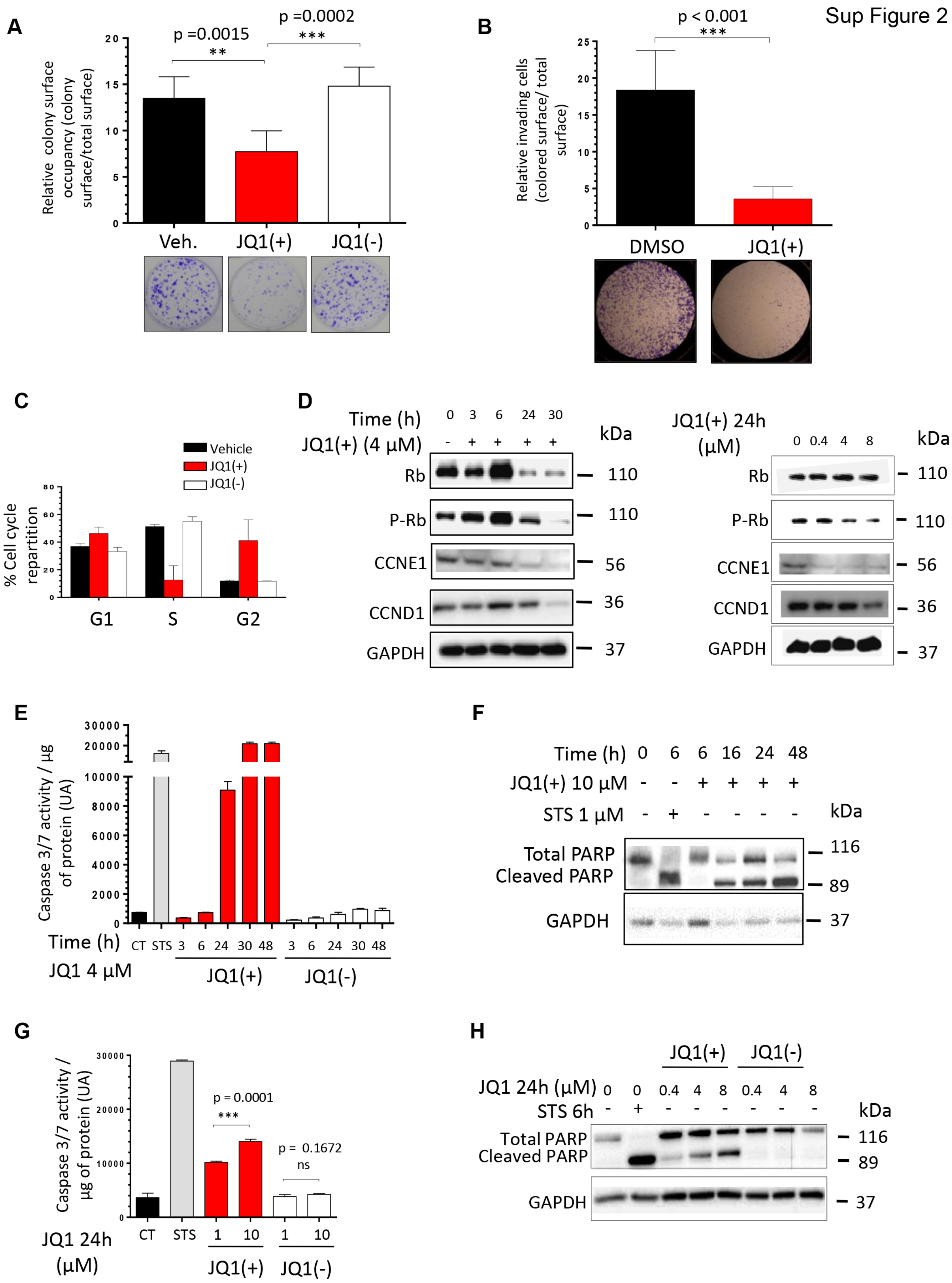

A
Relative colony surface occupancy (colony surface/total surface)
p =0.0015
**
p =0.0002
***
Veh.
JQ1(+)
JQ1(-)
B
Relative invading cells (colored surface/ total surface)
p < 0.001
***
DMSO
JQ1(+)
C
% Cell cycle repartition
Vehicle
JQ1(+)
JQ1(-)
G1
S
G2
D
Time (h)
0 3 6 24 30
JQ1(+) (4 µM)
- + + + +
kDa
Rb
P-Rb
CCNE1
CCND1
GAPDH
110
110
56
36
37
JQ1(+) 24h (µM)
0 0.4 4 8
E
Caspase 3/7 activity / µg of protein (UA)
Time (h)
CT STS 3 6 24 30 48 3 6 24 30 48
JQ1 4 µM
JQ1(+)
JQ1(-)
F
Time (h) 0 6 6 16 24 48
JQ1(+) 10 µM - - + + + +
STS 1 µM - + - - - -
Total PARP
Cleaved PARP
GAPDH
116
89
37
G
Caspase 3/7 activity / µg of protein (UA)
p = 0.0001
***
p = 0.1672
ns
JQ1 24h (µM)
CT STS 1 10 1 10
JQ1(+)
JQ1(-)
H
JQ1(+)
JQ1(-)
JQ1 24h (µM) 0 0 0.4 4 8 0.4 4 8
STS 6h - + - - - - - -
Total PARP
Cleaved PARP
GAPDH
116
89
37

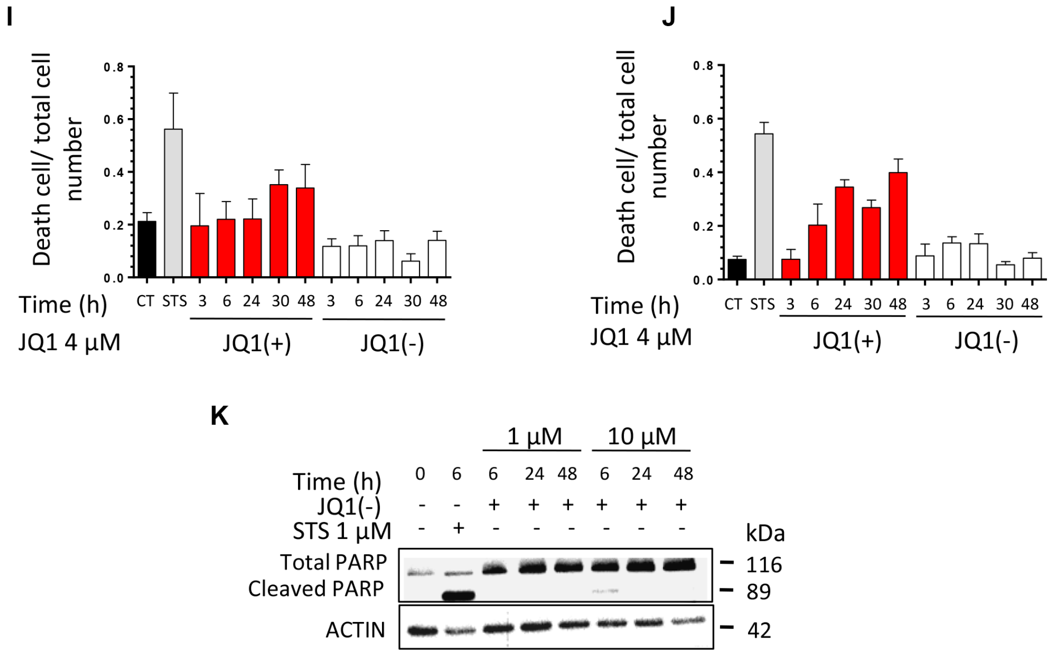

Supplementary Figure S2: JQ1 inhibits the clonogenicity, the migratory potential and induces both a G1-phase cell cycle arrest and the apoptosis of human Ewing Sarcoma cell lines. (**A**) The A673 Ewing Sarcoma cell line was plated at clonal density for colony numeration and treated with 4 µM JQ1(+) for 48 hours. Colony number was counted thanks to crystal violet staining performed after a 6-days incubation time and pictures of representative wells were taken. (**B**) The A673 cells were cultured or not in presence of 1 µM JQ1(+) during 24 hours and were then plated in Boyden Chambers, always in presence of JQ1(+) for additional 48 hours. A 1%/10% FBS-gradient was generated between the upper and the lower Chamber of the system, to promote the cell migration. At the end of the incubation time, the cells on the upper side of the Chamber were removed and those on the lower side of the Chamber were fixed with 10% Glutharaldeyde and stained with Crystal Violet. Pictures of the Chambers were taken and four different areas were arbitrary chosen to perform quantitative analyses. The ratio « colored surface/ total surface » was determined thanks to the ImageJ software. Representative pictures of the Boyden were chosen here. For all the Boyden Chambers experiments, error bars show the standard deviation for n = 8 measurements from representative experiments and a two-tailed paired Student's t-test was used to compare the different conditions. (**C**) The A673 Ewing Sarcoma cell line was treated with 1 µM JQ1(+) for 48 hours and the proportion of cells in G1, S, and G2 phase was determined by propidium iodide staining. (**D**) The A673 Ewing Sarcoma cell line was treated with 4 µM JQ1(+) for 3, 6, 24 or 30 hours and the cell cycle-related proteins Rb, phosphorylated-Rb, CCNE1and CCND1 expression levels were evaluated by Western blotting (left panel). The expression of the same proteins was assessed after treating or not the cells with JQ1(+) at 0.4, 4 or 8 µM during twenty-four hours (right panel). These experiments were repeated at least twice. Error bars show standard deviation for n = 3 measurements from representative experiments. (**E**) A673 cells were treated with 4 µM of JQ1(+) or JQ1(–) for 3, 6, 24, 30 or 48 hours, and the apoptosis was evaluated by dosage of the caspase 3/7 activities. (**F**) The A673 cell line was treated with 10 µM JQ1(+) for 6, 16, 24 or 48 hours and apoptosis was evaluated by cleaved poly (ADP-ribose) polymerase (PARP) level by Western blotting. (**G**) The apoptosis was evaluated by dosage of the caspase 3/7 activity in the A673 Ewing Sarcoma cell line after JQ1(+) or JQ1(–) treatment at 1 or 10 µM during 24 hours. (**H**) The same cell line was treated with 0.4, 4 or 8 µM JQ1(+) or JQ1(–) for 24 hours and apoptosis was evaluated by cleave dpoly (ADP-ribose) polymerase (PARP) level by Western blotting. For all the Western blotting experiments, the Glyceraldehyde-3-phosphate dehydrogenase was used as a loading control.These experiments were repeated at least twice. (**I**) The TC71 cell line and (**J**) the A673 one were treated with 4 µM of JQ1(+) or JQ1(–) for 3, 6, 24, 30 or 48 hours, the number of dead cells was evaluated by Trypan blu exclusion coloration. (**K**) A673 Ewing Sarcoma cell line was treated with 1 or 10 µM JQ1(–) for 6, 24 or 48 hours and apoptosis was evaluated by cleaved poly (ADP-ribose) polymerase (PARP) level by Western blotting. Actin was used as a loading control. For all the apoptosis-induction assessment assays, a 6 hours Staurosporine (STS) treatment at 1 µM is used as a positive control. For the cell-death-related experiments, error bars show standard deviation for n = 3 measurements from representative experiments. A two-tailed paired Student's t-test was used to compare the different conditions in these assays.

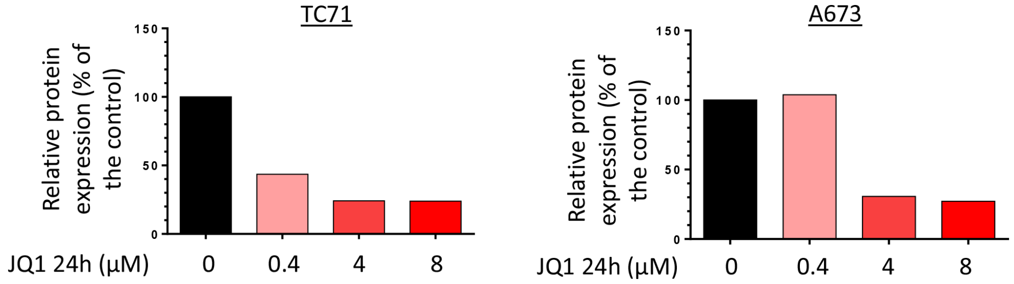

Supplementary Figure S3: JQ1(+) inhibits EWS-Fli1 expression and reduces the clonogenic capabilities of the Ewing Sarcoma cells whereas JQ1(–) does not. The TC71 (left panel) and A673 (right panel) Ewing Sarcoma cell lines were treated or not with 0.4, 4 or 8 µM JQ1(+) for 24 hours and the EWS-Fli1 expression level was evaluated by Western blotting. Glyceraldehyde-3-phosphate dehydrogenase was used as a loading control. Graphs show the relative quantization of EWS-Fli1 expression normalized on the Glyceraldehyde-3-phosphate dehydrogenase expression, in percentage of the control conditions.

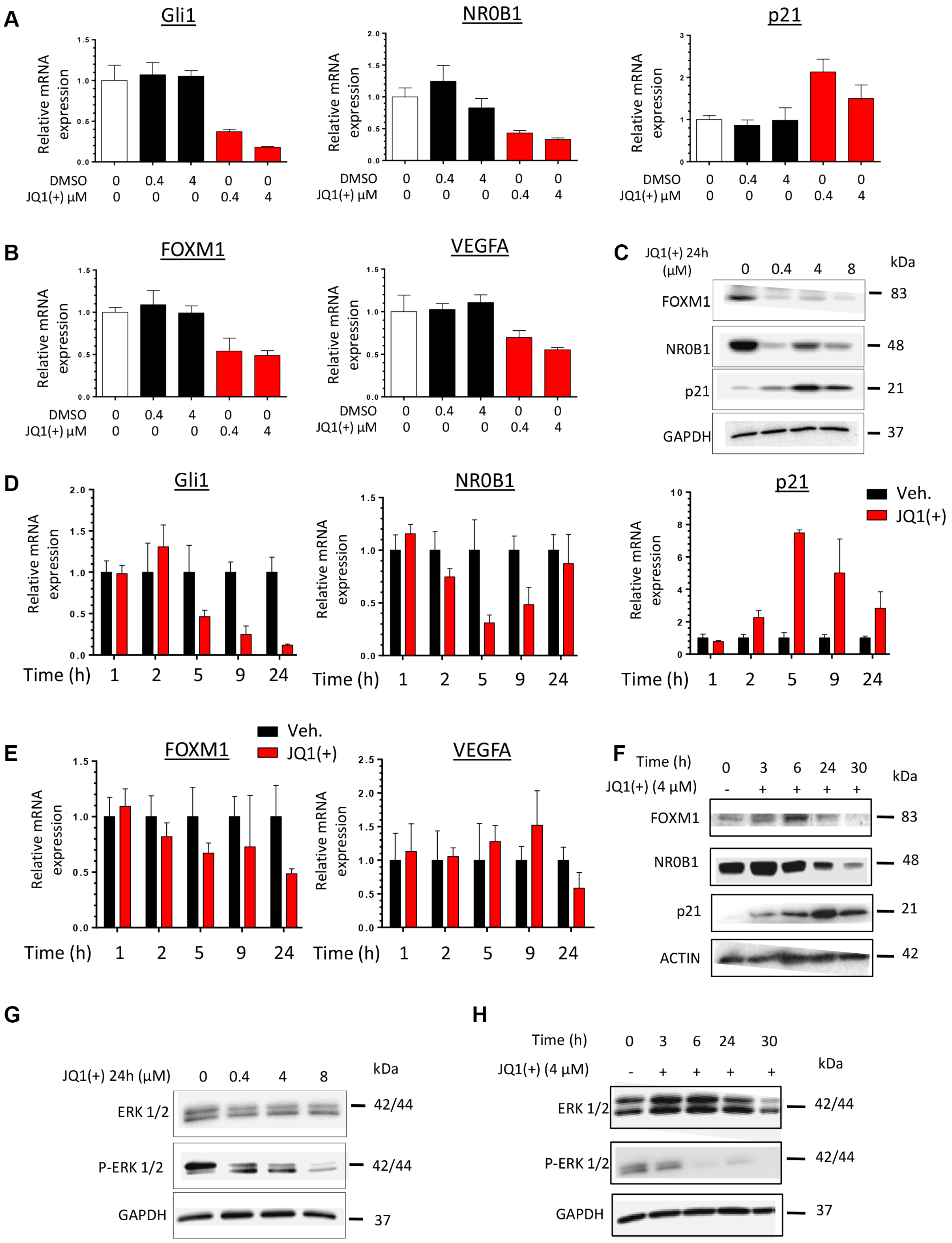
A
Gli1
NR0B1
p21
Relative mRNA expression
DMSO 0 0.4 4 0 0
JQ1(+) µM 0 0 0 0.4 4
B
FOXM1
VEGFA
Relative mRNA expression
DMSO 0 0.4 4 0 0
JQ1(+) µM 0 0 0 0.4 4
C
JQ1(+) 24h (µM) 0 0.4 4 8
kDa
FOXM1 83
NR0B1 48
p21 21
GAPDH 37
D
Gli1
NR0B1
p21
Relative mRNA expression
Time (h) 1 2 5 9 24
Veh.
JQ1(+)
E
FOXM1
VEGFA
Veh.
JQ1(+)
Relative mRNA expression
Time (h) 1 2 5 9 24
F
Time (h) 0 3 6 24 30
JQ1(+) (4 µM) - + + + +
kDa
FOXM1 83
NR0B1 48
p21 21
ACTIN 42
G
JQ1(+) 24h (µM) 0 0.4 4 8
kDa
ERK 1/2 42/44
P-ERK 1/2 42/44
GAPDH 37
H
Time (h) 0 3 6 24 30
JQ1(+) (4 µM) - + + + +
kDa
ERK 1/2 42/44
P-ERK 1/2 42/44
GAPDH 37

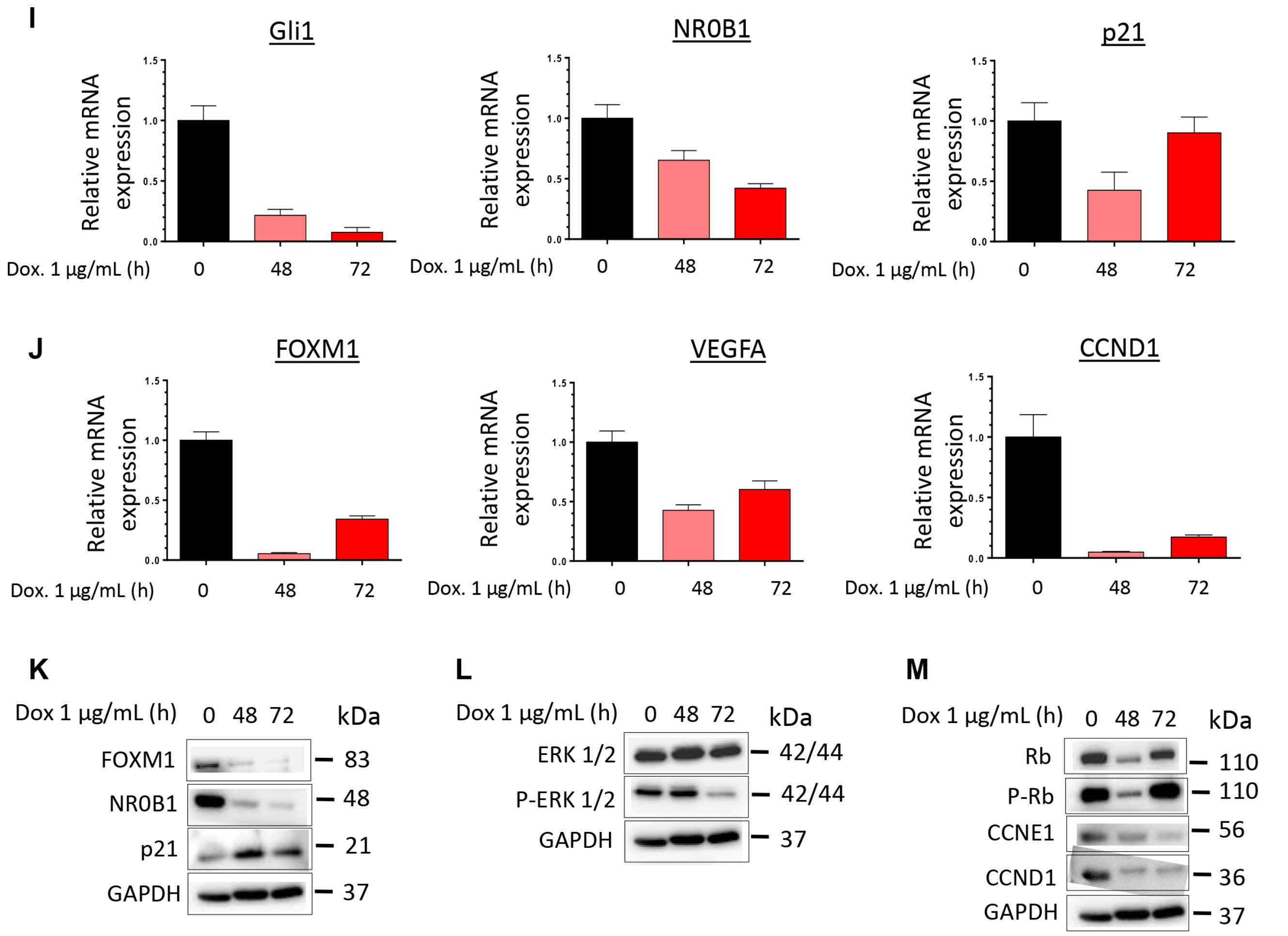

Supplementary Figure S4: JQ1(+) treatment in Ewing Sarcoma cells induces a dose- and a time-dependant EWS-Fli1-target-genes expression modulation and also modulates pathways under the control of EWS-Fli1 whereas JQ1(–) does not. EWS-Fli1 knock-down with shRNA mimics the BET bromodomain inhibition's effects of JQ1(+) in Ewing Sarcoma. (**A**) qRT–PCR for EWS-Fli1 direct-target genes (Gli1, NR0B1 and p21) and (**B**) EWS-Fli1 indirect-target genes (FOXM1 and VEGFA) in DMSO or JQ1(+)-treated Ewing Sarcoma TC71 cell line during 24 hours, at 0.4 and 4 µM JQ1(+). (**C**) FOXM1-, NR0B1- and p21-EWS-Fli1-target gene expression was evaluated at protein level by Immunoblotting in the TC71 Ewing Sarcoma cell line after JQ1(+) treatment at 0.4, 4 or 8 µM during 24 hours.Glyceraldehyde-3-phosphate dehydrogenase was used as a loading control. (**D**) qRT–PCR for EWS-Fli1 direct-target genes (Gli1, NR0B1and p21) and (**E**) EWS-Fli1 indirect-target genes (FOXM1 and VEGFA) in DMSO or JQ1- (+)-treated Ewing Sarcoma TC71 cells at 4 µM during 1, 2, 5, 9, and 24 hours. (**F**) FOXM1-, NR0B1- and p21-EWS-Fli1-target gene expression was evaluated at protein level by Immunoblotting in the same cell line after JQ1(+) treatment or not, during 3, 6, 24, or 30 hours. Actin was used as a loading control. (**G**) ERK ½ phosphorylation level was evaluated by Immunoblotting in the TC71 Ewing Sarcoma cell line after a 24 hours JQ1(+) treatment at 0.4, 4 or 8 µM or after a 4 µM JQ1(+) treatment during 3, 6, 24, or 30 hours (**H**). Glyceraldehyde-3-phosphate dehydrogenase was used as a loading control. (**I**) The expression of EWS-Fli1 direct-target genes (Gli1, NR0B1 and p21) and **(J)** EWS-Fli1 indirect-target gene (FOXM1, VEGFA and CCND1) was assessed by qRT–PCR in the ASP14 cell line treated or not with 1 µg/mL Doxycycline for 48 or 72 hours, inducing or not the transcription of the shEWS-Fli1. (**K**) FOXM1-, NR0B1- and p21-EWS-Fli1-target genes expression was evaluated at protein level by Immunoblotting in the ASP14 cell line in the same conditions as in (J). (**L**)ERK ½ phosphorylation level was evaluated by Immunoblotting in the same cell line and in the same conditions as in (J). (**M**) The cell cycle-related proteins Rb, phosphorylated-Rb, CCNE1 and CCND1 expression levels were evaluated by Western blotting in the same conditions as described in (J). These Western blots were performed at least twice and representative blots are presented. Glyceraldehyde-3-phosphate dehydrogenase was used as a loading control. For all the qRT-PCR, GAPDH and B2M are used as housekeeping genes and error bars show standard deviation for n = 3 measurements from representative experiments.

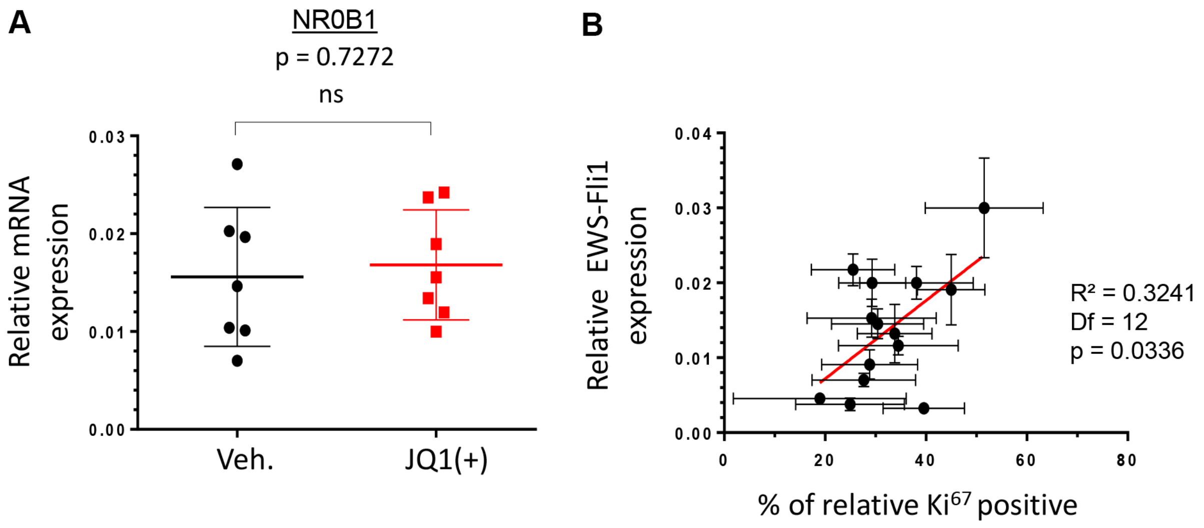

Supplementary Figure S5: JQ1(+) has no effect on the NR0B1 direct EWS-Fli1 target-gene expression in the tumor biopsies of TC71 Ewing Sarcoma *in vivo* model but induces an EWS-Fli1 down-regulation both at mRNA and protein level. (**A**) NR0B1 expression was evaluated in tumor tissues, after RNA extraction, by qRT–PCR. Unpaired two-tailed *t*-test was used to compare the gene expression between groups. GAPDH and B2M are used as housekeeping genes and error bars show standard deviation for n = 3 measurements from representative experiments. (**B**) Correlation between Ki^{67} marker and EWS-Fli1 expression in the same tumor tissues as presented in (A), assessed by immunohistochemical staining and by qRT–PCR and tested by the *Pearson product-moment correlation test*.
